# Cystine–glutamate antiporter deletion accelerates motor recovery and improves histological outcomes following spinal cord injury in mice

**DOI:** 10.1038/s41598-021-91698-y

**Published:** 2021-06-09

**Authors:** Lindsay Sprimont, Pauline Janssen, Kathleen De Swert, Mathias Van Bulck, Ilse Rooman, Jacques Gilloteaux, Ann Massie, Charles Nicaise

**Affiliations:** 1 URPhyM – NARILIS, Université de Namur, Rue de Bruxelles 61, 5000 Namur, Belgium; 2 Neuro-Aging & Viro-Immunotherapy, Center for Neurosciences (C4N), Vrije Universiteit Brussel, Brussels, Belgium; 3 Oncology Research Center, Vrije Universiteit Brussel, Brussels, Belgium; 4 Department of Anatomical Sciences, St George’s University School of Medicine, Newcastle upon Tyne, UK

**Keywords:** Transporters in the nervous system, Spinal cord diseases

## Abstract

xCT is the specific subunit of System xc-, an antiporter importing cystine while releasing glutamate. Although xCT expression has been found in the spinal cord, its expression and role after spinal cord injury (SCI) remain unknown. The aim of this study was to characterize the role of xCT on functional and histological outcomes following SCI induced in wild-type (xCT+/+) and in xCT-deficient mice (xCT−/−). In the normal mouse spinal cord, *slc7a11*/xCT mRNA was detected in meningeal fibroblasts, vascular mural cells, astrocytes, motor neurons and to a lesser extent in microglia. *slc7a11*/xCT gene and protein were upregulated within two weeks post-SCI. xCT−/− mice recovered muscular grip strength as well as pre-SCI weight faster than xCT+/+ mice. Histology of xCT−/− spinal cords revealed significantly more spared motor neurons and a higher number of quiescent microglia. In xCT−/− mice, inflammatory polarization shifted towards higher mRNA expression of *ym1* and *igf1* (anti-inflammatory) while lower levels of *nox2* and *tnf-a* (pro-inflammatory). Although astrocyte polarization did not differ, we quantified an increased expression of *lcn2* mRNA. Our results show that *slc7a11*/xCT is overexpressed early following SCI and is detrimental to motor neuron survival. xCT deletion modulates intraspinal glial activation by shifting towards an anti-inflammatory profile.

## Introduction

Minutes to days following spinal cord injury (SCI), hemorrhage, ion dysregulation, production of reactive oxygen species and excitotoxicity are amongst the pathological events responsible for the propagation of damage to spared spinal tissue surrounding the epicenter. During this so-called secondary injury, the volume of the initial trauma expands and leads to additional tissue loss, and therefore worsens the functional deficits^1^. Within minutes post-SCI, abnormal-to-toxic levels of glutamate are reached in the extracellular space and this can persist for more than a week^2–5^. Beside a focal glutamate rise, glutamate homeostasis is also disturbed at remote lesioned sites, likely via spreading mechanisms involving activated glial cells^6^. Oligodendrocytes and motor neurons are amongst the most vulnerable cells to glutamate excitotoxicity as they have a high expression of amino-3-hydroxy-5-methylisoazol-4-propionate receptor and N-methyl-D-aspartate receptor respectively, and they are endowed with limited calcium buffering abilities^3,7^.

In the spinal cord, glutamate is the most represented neurotransmitter whose release is timely regulated, which supports an efficient and transient neurotransmission, while avoiding excessive extracellular glutamate concentrations. In physiological conditions, glutamate is maintained at very low levels in the synaptic cleft and in the extrasynaptic spaces, through a variety of membranous transporters on neuronal or non-neuronal cells. High affinity excitatory amino-acids transporters (EAATs) are expressed by astrocytes and account for up to 90% of synaptic glutamate re-uptake^8^. Emerging as another player in glutamate homeostasis, System xc- has been identified as the major source of glutamate released in the extrasynaptic spaces of the hippocampus and the striatum^9–12^. System xc− is a Na+ -independent electroneutral antiporter composed of xCT specific subunit acting as the cysteine–glutamate antiport and bound to the 4F2 heavy chain subunit involved in its membrane trafficking^13,14^. xCT protein is encoded by the *slc7a11* gene located on chromosome 3 in mice and on chromosome 4 in humans. xCT functions to export one molecule of glutamate in exchange for one cystine imported, in a 1:1 ratio. Cystine is further reduced intracellularly into cysteine that serves as a building block for glutathione (GSH)^15,16^. The cellular expression of xCT is still a matter of debate; some authors found it in microglia^17–19^, while others in astrocytes or oligodendrocytes^20–23^, but insofar not in neurons^19,22^. The activity of xCT is induced in presence of reactive oxygen species or inflammatory stimuli^24–26^. This suggests that an enhanced activity, while beneficially boosting GSH production, can at the same time lead to inopportune glutamate release. Several lines of evidence argue towards an active contribution of xCT in the progression of spinal cord disorders. For instance, xCT protein expression is highly upregulated in the spinal tissue of patients suffering from multiple sclerosis (MS) or amyotrophic lateral sclerosis (ALS)^27,28^. Moreover, xCT deficiency produces significant improvements of disease outcomes in MS^28,29^ and ALS^27^ animal models, suggesting that inhibition of glutamate release via System xc− , or another mechanism, would somehow confer neuroprotection.

xCT being a cystine–glutamate antiporter, we hypothesized that it could contribute to glutamate excitotoxicity, alleviate oxidative stress, or even modulate inflammation following spinal cord injury (SCI). To elucidate the role of System xc-, wild-type (xCT+/+) and xCT knock-out (xCT−/−) mice were subjected to cervical contusive SCI. In both injured groups of animals, we assessed functional motor outcomes, histological findings, completed by molecular markers of glial activation and inflammation polarization. In addition, we provide new data on cellular distribution of *slc7a11*/xCT mRNA in the normal and injured mouse spinal cord.

## Results

### slc7a11/xCT is mostly expressed in meningeal cells and spinal astrocytes, and is upregulated following SCI

**Figure 1:**
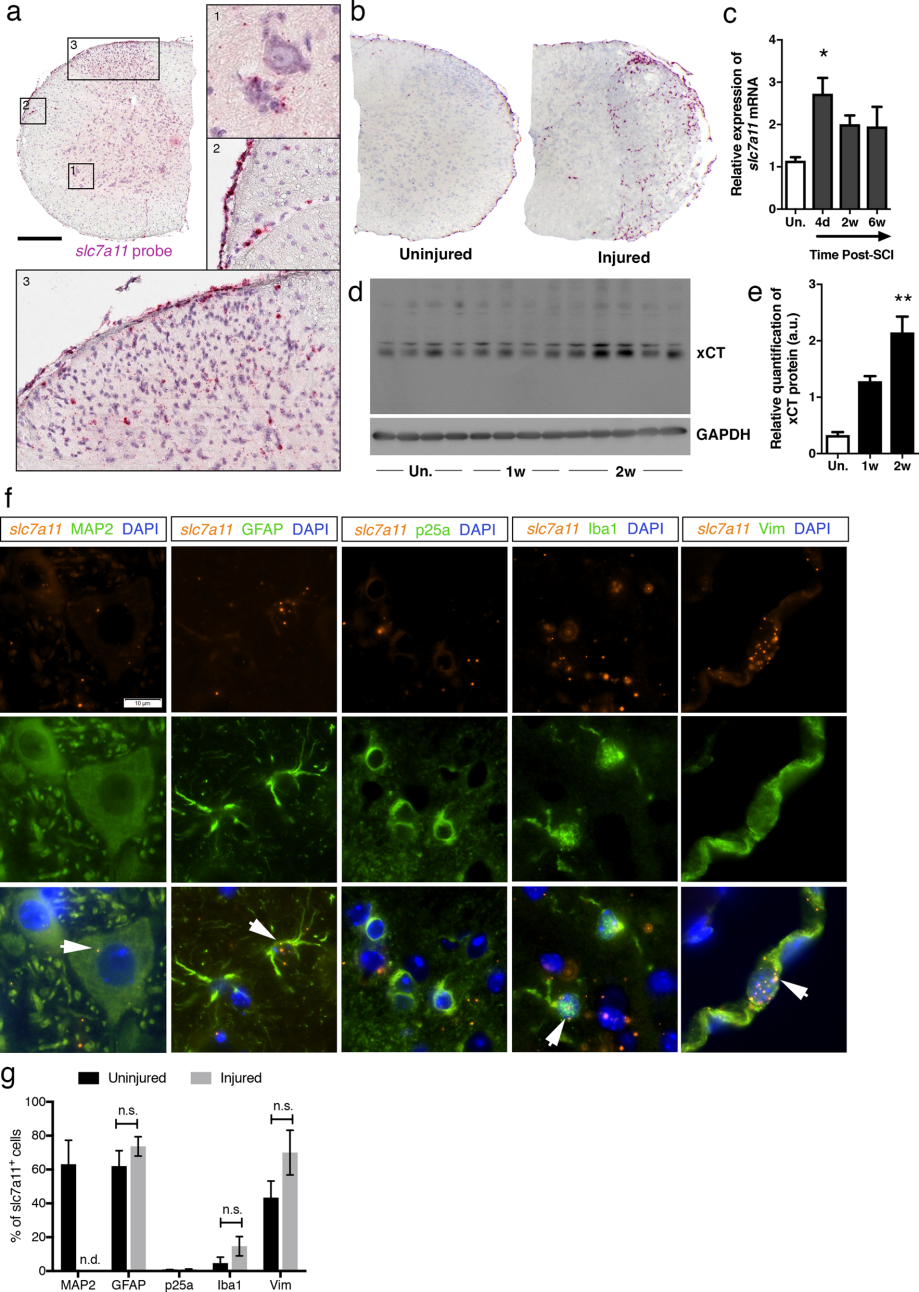
Cellular *slc7a11*/xCT gene expression in the mouse spinal cord. A probe targeting *slc7a11* mRNA was applied onto uninjured and injured spinal cords. The chromogenic signal of the in situ hybridization was revealed as pink deposits, whose size was proportional to the local amount of transcripts (**a**,**b**). In the normal spinal cord, *slc7a11*/xCT mRNA expression was predominantly detected in nervous cells satellite to motor neurons (**a**, inset 1), in the meningeal layer and along the wall of the perforating blood vessels (**a**, inset 2), and in the neuropil around the dorsal horn neurons (**a**, inset 3). A higher abundance of transcripts was detected in the lesion area from spinally-injured mice (**b**). qRT-PCR confirmed the overall increased expression of *slc7a11*/xCT mRNA in injured spinal cords, significantly peaking at 4 days post-injury (**c**) (*p < 0.05, Kruskal–Wallis ANOVA test, Un. n = 3; 4d n = 8; 2w n = 3; 6w n = 3). Immunoblot showing xCT and GAPDH protein expression at different timings post-SCI (**d**). Full length blot of xCT detection is displayed where xCT specific band was detected at approximately 40 kDa. GAPDH was used as loading control. Uncropped images are available in Supplementary Fig. 2. Compared to uninjured tissue (Un.), xCT was upregulated at the protein level at two weeks post-injury (**e**) (*p < 0.01, Kruskal–Wallis ANOVA test, Un. n = 4; 1w n = 4; 2w n = 5). Fluorescent *slc7a11 *in situ hybridization (orange) and immunofluorescent co-labeling (green) of GFAP, Iba1, p25a, MAP2 and vimentin in uninjured spinal cords (**f**). The majority of slc7a11 + cells were co-labeled with GFAP, vimentin, MAP2 and Iba1 (white arrows) (**g**). The nuclei were counterstained with DAPI (blue). *slc7a11* expression was rarely detected in p25a + oligodendrocytes. Injury did not significantly modify the cellular expression profile of *slc7a11* (*p* = n.s., Mann–Whitney test, n = 3 spinal slices in each group). Data are expressed as mean +/− SEM. Scale bars = 250 µm in a and 10 µm in f. Abbreviations : n.d. = not done, as MAP2 + motor neurons were completely lost at the injury epicenter.

The expression of *slc7a11*/xCT mRNA was investigated using in situ hybridization (*ish*) at several cervical levels of the adult C57BL/6J mouse spinal cord. The results consistently showed chromogenic deposits in the normal spinal cord with a preferential distribution throughout the gray matter and the pia mater (Fig. 1a, Supplementary Fig. 1a). Spinal tissue from xCT−/− mouse was used as negative control; absence of *slc7a11* signal demonstrated the specificity of the probe (Supplementary Fig. 1b). Mouse genotype (+/+ , + /− or −/−) was confirmed by PCR and real-time quantitative PCR (Supplementary Fig. 1c). In the gray matter of xCT+/+ mice, *slc7a11* mRNA labeling was detected in the neuropil; in the ventral horns as wells as in the intermediate and dorsal layers, mostly scattered between neuronal cell bodies (Fig. 1a, insets 1 and 3). Rexed laminae I, II and III were particularly enriched in cells bearing *slc7a11* mRNA (Fig. 1a, inset 3). Cells that belonged to the meningeal layers also showed strong *slc7a11* probe labeling as well as mural cells along the perforating blood vessels (Fig. 1a, inset 2). Following unilateral spinal cord injury, the signal density for *slc7a11* probe drastically increased compared to the contralateral side or compared to an uninjured spinal cord (Fig. 1b). The overall amount of *slc7a11* mRNA in the lesion was 2.7 times higher within the fourth day post-injury (Kruskal–Wallis ANOVA; uninjured vs. 4 days post-SCI; *p* = 0.0413), with a tendency towards a plateau at 2- and 6-weeks post-SCI (Fig. 1c). Using anti-xCT antibody, we showed an upregulation of spinal xCT protein expression at the second week post-SCI (Kruskal–Wallis ANOVA; uninjured *vs.* 2 weeks post-SCI; *p* = 0.0058) (Fig. 1d, e). Protein extracts from xCT−/− spinal cords were used as negative control to verify the specificity of the antibody. Although many studies rely on a predicted molecular weight at 55.5 kDa, a band between 34 and 43 kDa was reliably detected in all CNS samples from xCT+/+ mice, that was absent from xCT−/− matched tissue (Supplementary Fig. 1d). Extra bands at higher molecular weight (around 130 kDa) were also revealed; further investigations did not allow to conclude on the nature of the bands. Next, the cellular expression of *slc7a11* mRNA was investigated on spinal slices using double labeling for astroglial (GFAP), microglial (Iba1), oligodendroglial (p25alpha), neuronal cells (MAP2) and intermediate filaments (vimentin). *Slc7a11* + cells were co-labeled with GFAP, MAP2 and Iba1 at various degrees, but very rarely with p25alpha (Fig. 1f). Quantification of double-labeled cells in a healthy spinal cord showed that the vast majority of *slc7a11* + cells were immunoreactive for GFAP (61%), vimentin (43%), MAP2 (64%) and to a lesser extent for Iba1 (4%) (Fig. 1g). Unlike GFAP being almost exclusively expressed by astrocytes, vimentin was also found in meningeal fibroblasts. Both cell types showed significant *slc7a11* probe co-labeling (Fig. 1f). One week after SCI, the proportion of *slc7a11* + cells co-labeled with GFAP, vimentin, p25alpha or Iba1 did not significantly change (Fig. 1g).

### Spinally-injured xCT−/− mice had a faster body weight and motor recovery than wild-type littermates

**Figure 2:**
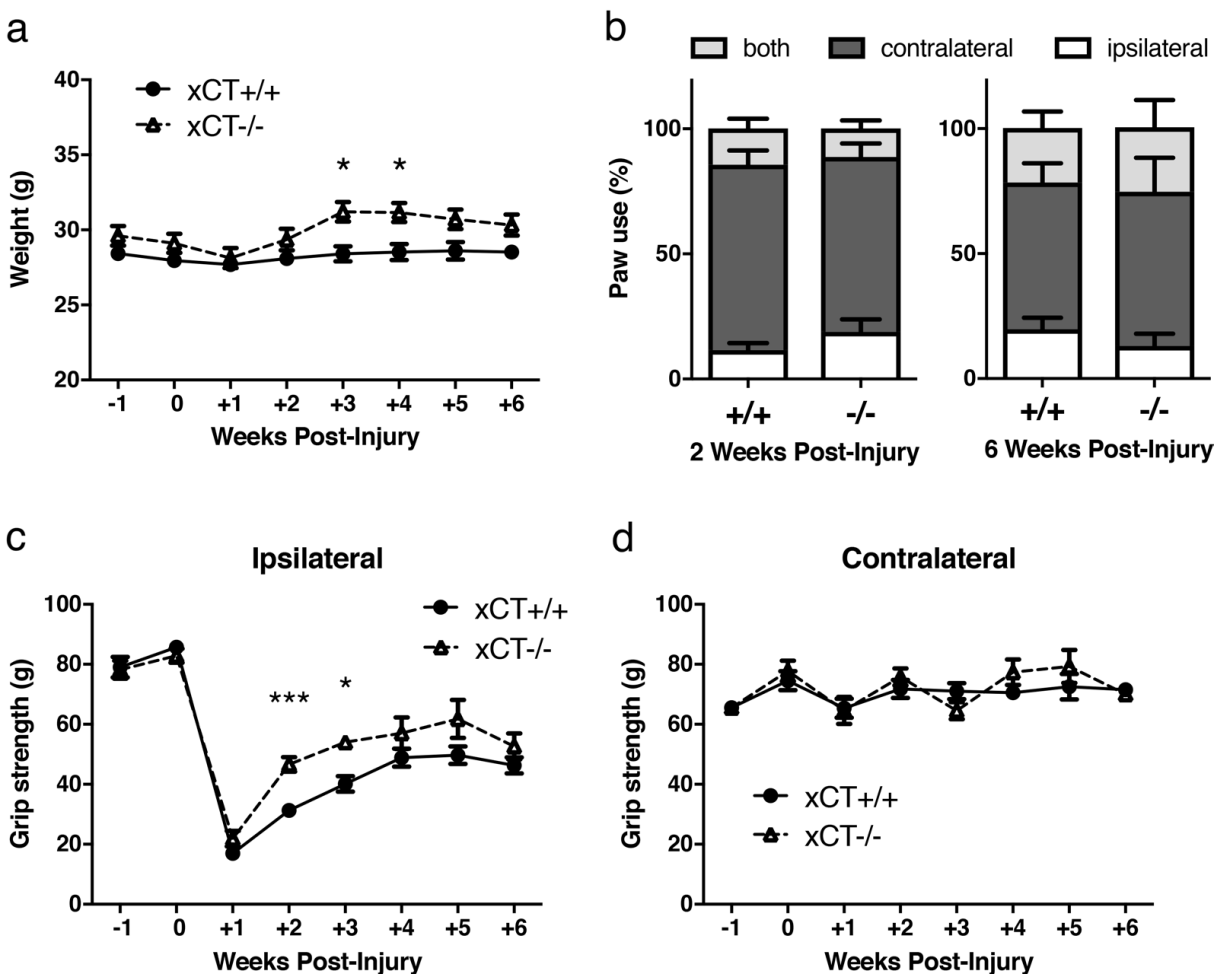
Evolution of weight and motor function in injured xCT+/+ and xCT−/− at different timing post-injury. xCT−/− mice recovered pre-SCI weight and gained weight faster than xCT+/+ between 3 and 4 weeks post-injury (**a**). Use of forelimbs, either ipsilateral or contralateral (or both) to the spinal lesion, was not different between injured xCT+/+ and xCT−/− mice (**b)**. Grip strength test showed a better recovery of the ipsilateral grip strength for xCT−/− mice than for xCT+/+ mice at 2- and 3- weeks post-injury (**c**). Both groups of mice recovered similarly at later time points. The contralateral grip strength was not impaired by the unilateral spinal cord injury (**d**). *p < 0.05 and ***p < 0.001, two-way ANOVA followed by Sidak’s multiple comparisons, n = 10–12 mice/group between -1 and 2 weeks post-injury, n = 4–8 mice/group between 3 and 6 weeks post-injury. Data are expressed as mean +/− SEM.

To elucidate the role of System xc− following SCI, wild-type (xCT+/+) and knock-out (xCT−/−) mice were subjected to unilateral cervical contusive injury. To follow the evolution of forelimb motor function, both groups of injured mice were monitored for 6 weeks post-SCI using weight, paw preference test, and grip strength testing. Within the first week after SCI, the weight of mice dropped about 2 g, and progressively returns to pre-SCI basal level the following weeks (Fig. 2a). Injured xCT−/− mice regained weight faster at 3- and 4- weeks post-SCI than xCT+ /+ mice (two-way ANOVA; F(1,142) = 36.14 for genotype comparison xCT+/+ vs. xCT−/−; *p* = 0.0141 and *p* = 0.0264 at 3- and 4- weeks post-SCI respectively). During exploration in the cylinder paw preference test, there was an impairment in the animals' ability to use the ipsilateral forepaw after SCI. However, no difference in forepaw use between both genotypes could be found during the entire follow-up period of 6 weeks (Fig. 2b). Compared to pre-SCI condition, both groups undergoing contusion SCI showed an evident drop in their ipsilateral forelimb grip strength the first week post-injury (Fig. 2c). The injured xCT−/− mice subsequently recovered a stronger grip force in the affected forelimb than xCT+/+ littermates (two-way ANOVA; F(1,134) = 20.97 for genotype comparison xCT+/+ vs. xCT−/−; *p* = 0.0004 and *p* = 0.0401 at 2- and 3- weeks post-SCI respectively). Contralateral forelimb score was not affected at any time point, consistent with a unilateral nature of the lesion (Fig. 2d).

### Spinal GSH levels and oxidized proteins are similar in injured xCT+/+ and xCT−/−

**Figure 3:**
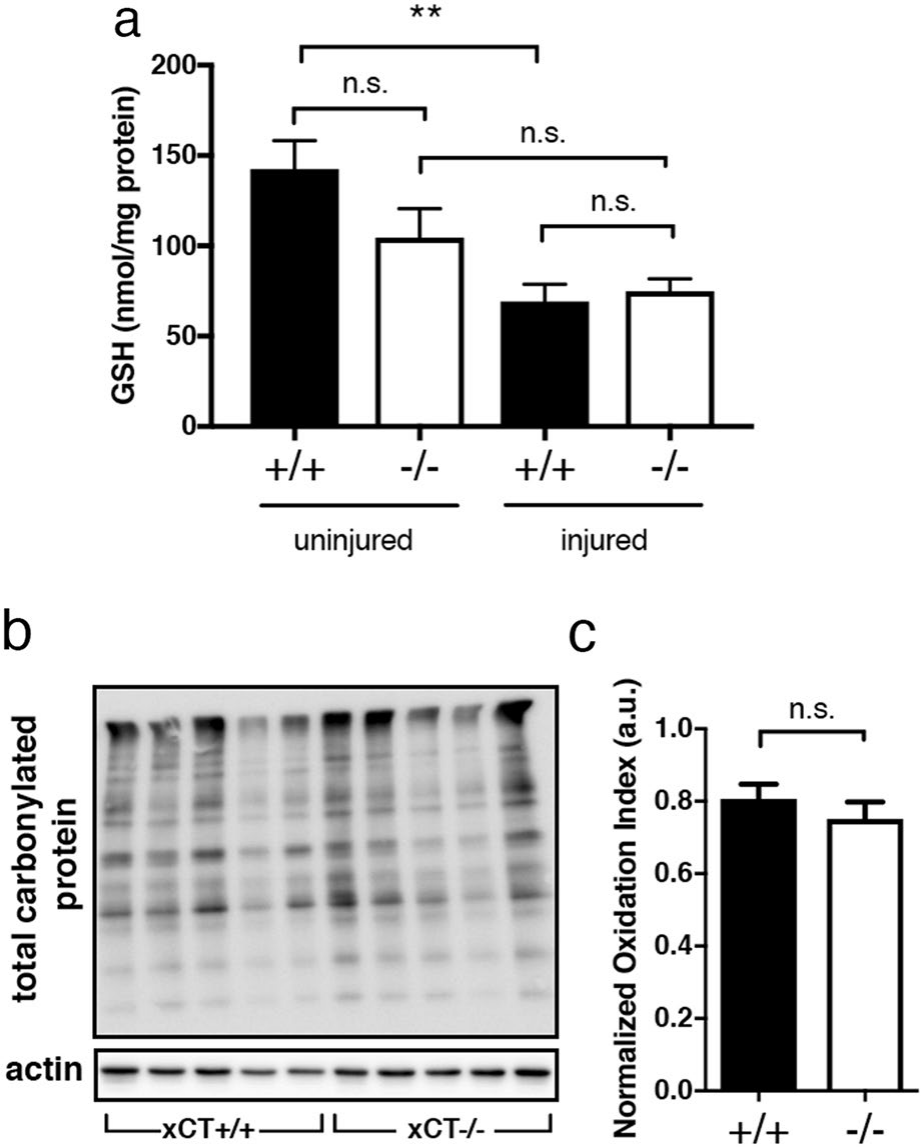
Glutathione levels and oxidative stress-related markers following SCI. Spinal GSH content in pre-injury and injury groups. Injured xCT+/+ and xCT−/− mice had equal amount of GSH two weeks after SCI (**a**). GSH levels are significantly decreased after injury in xCT+/+ mice. **p < .01, two-way ANOVA followed by Sidak multiple comparisons test, n = 5–7 mice/group. Protein oxidation detection kit was used to quantify the amount of carbonylated proteins in the spinal cord at 2 weeks post-injury in injured xCT+/+ and xCT−/− mice (**b**). When normalized to actin, the oxidation index (a.u.) did not show any significant difference between groups (**﻿c**). Mann–Whitney test, n = 5 mice/group. Data are expressed as mean +/− SEM.

As System xc− acts as an importer of cystine across the membrane and is as such involved in the replenishment of intracellular glutathione, we sought to know to what extent the deletion of the xCT subunit would alter GSH production. In absence of injury, there was not significant difference in the amount of GSH in xCT−/− spinal cords compared to xCT+/+ tissues (two-way ANOVA; F(1,19) = 1.459 for genotype comparison xCT+/+ vs. xCT−/−; *p* = 0.1077) (Fig. 3a). This suggests that xCT−/− mice are still able to build GSH and, in a way, compensate for the loss of cystine import, through alternative pathways. As measured at two weeks, SCI induced a significant depletion of spinal GSH levels in injured xCT+/+ mice (two-way ANOVA; F(1,19) = 14.74 uninjured *vs.* injured; *p* = 0.0011), that yielded the same magnitude in the xCT−/− group. GSH content was therefore similar in both injured groups following SCI and GSH levels per se cannot account for the better early recovery observed in xCT−/− mice. Formation of carbonylated proteins has been described following SCI, consequently to oxidative stress during the secondary phase of SCI. Quantitative assessments of total carbonylated proteins, as referred as “oxidation index”, showed variability within a same experimental group and did not reveal any difference between injured xCT+/+ and xCT−/− tissues (Fig. 3b, c).

### The number of spared motor neurons is greater in injured xCT−/− spinal cords

**Figure 4:**
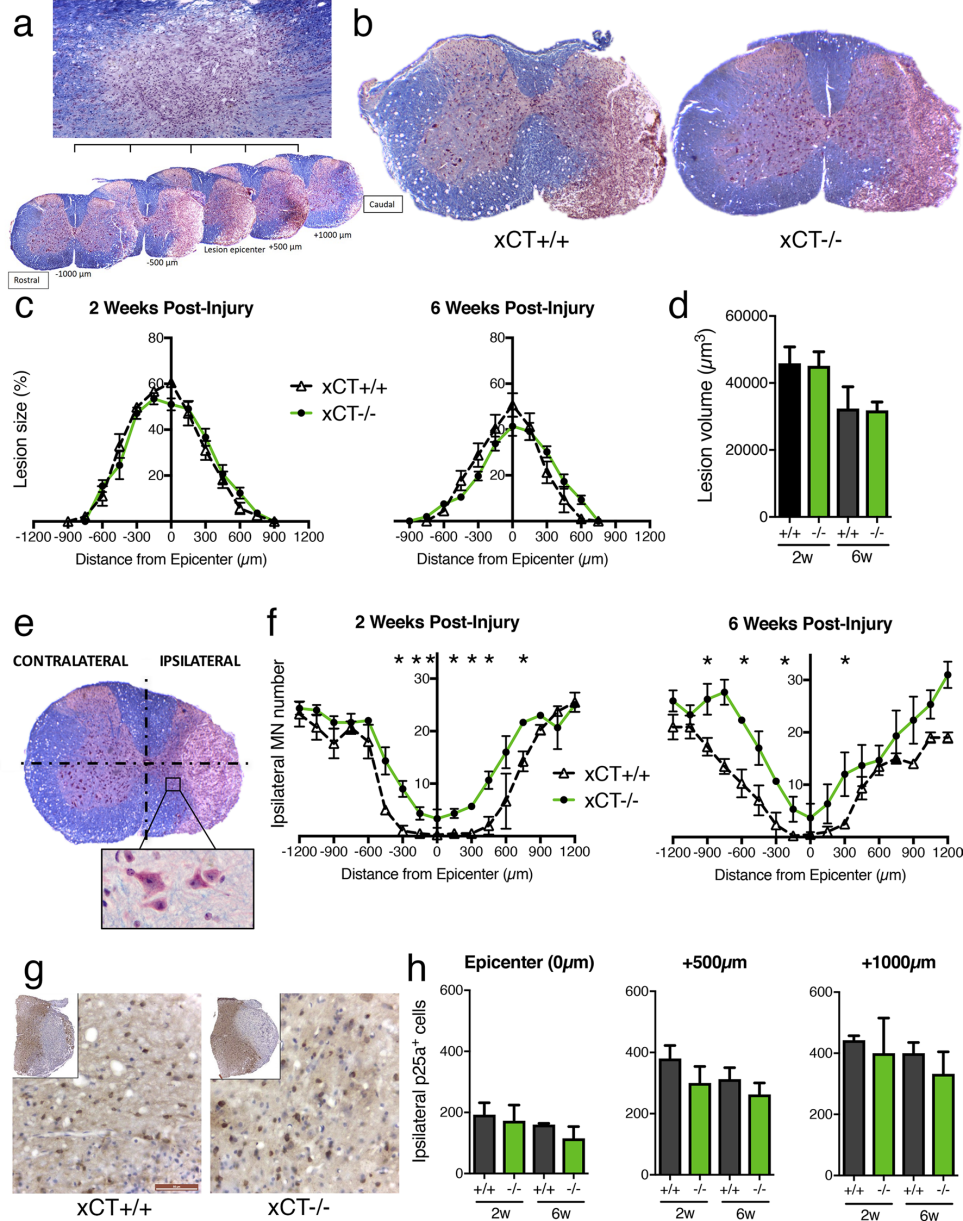
Quantification of lesion size, motor neuron and oligodendrocyte number in injured xCT+/+ and xCT−/− spinal cords. The diagram depicts how the unilateral lesion extends rostro-caudally from  − 1000 to + 1000 µm around the epicenter (**a**). The epicenter (set at 0 µm) was defined as the spinal level where the area of lesioned tissue is maximal. Representative micrographs of the lesion epicenter is shown for xCT+/+ (left) and xCT−/− (right) mice (**b**). Quantification of lesioned area (in percentage of ipsilateral hemicord) or lesion rostro-caudal extension did not show any difference between groups at 2 or 6 weeks post-injury (**c**). Quantification of lesion volume did not show any difference between groups at 2 or 6 weeks post-injury (**d**). As depicted, the number of spared motor neurons was counted in the inferior quadrants of both contralateral and ipsilateral sides, including the ventral horn and part of the intermediate column (**e**). For some distances along the lesion, ipsilateral motor neuron number was significantly higher in injured xCT−/− mice than in the xCT+/+ mice, at both 2 and 6 weeks post-injury (**f**). Data are expressed as mean +/− SEM. *p < 0.05, Mann–Whitney test, n = 3–4 mice/group. Using anti-p25a immunohistochemistry, the number of spared oligodendrocytes was evaluated in the injured xCT+/+ and xCT−/− hemicords, at three distances from the epicenter, 0 µm, + 500 µm and + 1000 µm (**g**,**h**). two-way ANOVA followed by Sidak multiple comparisons test, n = 3–4 mice/group. Data are expressed as mean +/− SEM. Scale bars = 50 µm in panel g.

In order to understand why xCT-deficient mice recovered earlier following SCI, we sought to correlate functional findings with histological outcomes. Occurring in hours-to-days after the initial traumatic contusion, the SCI lesion extends away from the epicenter (Fig. 4a) in a rostro-caudal fashion. The lesion is characterized by a disruption of spinal cord architecture, with a loss of neuronal cell bodies, demyelination or damages of nerve fibers, infiltration by immune cells and activation of glial cells. Lesion area was first analyzed at different distances along the cervical spinal cord in injured xCT+/+ and xCT−/− mice (Fig. 4b). Neither 2 weeks nor 6 weeks post-SCI did the lesion surface (Fig. 4c) or lesion volume (Fig. 4d) differ between both genotypes. In order to count the motor neuron cell bodies ipsilateral to the lesion, each spinal slice has been divided into 4 quadrants with the center of the central canal as the origin of the reference system. Counts were restricted to those cells that met the criteria of motor neurons, as stated in the Methods (Fig. 4e, inset). Compared with the xCT+/+ mice, injured xCT−/− mice showed an increased number of ipsilateral spared motor neurons at multiple distances from the lesion epicenter at both 2 and 6 weeks (Mann–Whitney test; *p* < 0.05 at multiple distances) (Fig. 4f). The number of motor neurons contralateral to the lesion did not differ between genotypes.

For further analysis of glial cell number, the lesion epicenter (0 µm) and two regions caudal to it (respectively at +500 and +1000 µm distances) were considered. Like motor neurons, oligodendrocytes are likely to undergo cell death at the lesion epicenter and their loss can be correlated to functional motor impairment. Therefore, the number of oligodendrocytes was investigated using anti-p25a immunohistochemistry (Fig. 4g). As expected, a drastic loss of p25a immunoreactivity was observed in the grey and white matter at 2 weeks post-SCI in both groups (inset in Fig. 4g). The quantification of p25a + cells did not reveal any difference in the number of oligodendrocytes (Fig. 4h), even at later time points when oligodendrocytes—or their precursors—are supposed to have started the remyelination process.

### Reactive astrogliosis is not significantly impacted in injured xCT−/− mice

**Figure 5:**
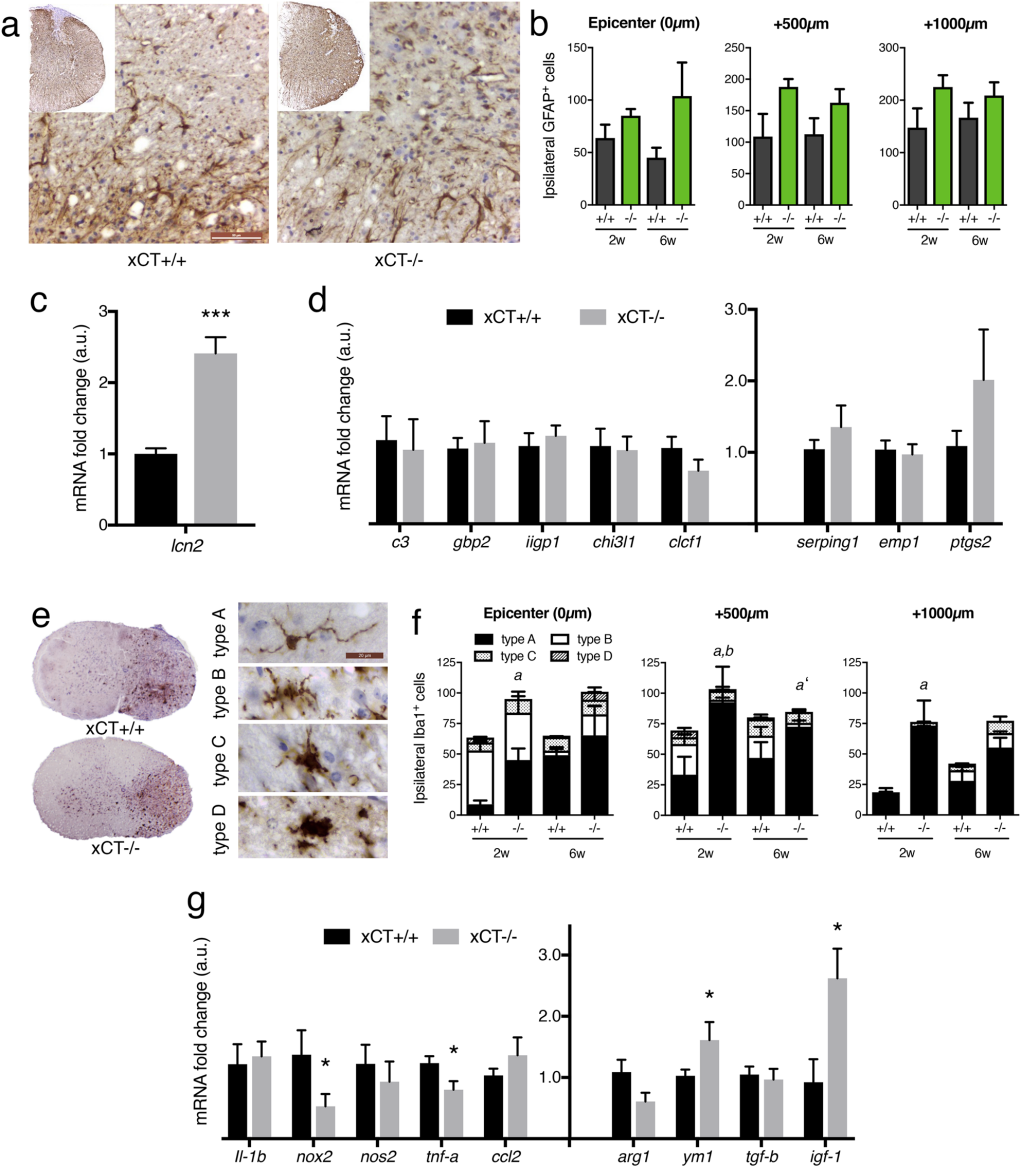
Assessment of astrocyte and microglia activation in injured xCT+/+ and xCT−/− spinal cords. Spinal cords from both genotypes were immunostained with GFAP antibody. Representative GFAP immunolabeling at the epicenter at 2 weeks post-injury for both genotypes (**a**). The counting of GFAP-positive cells was performed in the ipsilateral hemicord at epicenter, and + 500 µm and + 1000 µm caudal to the epicenter (**b**). No significant change in astrocyte number was observed. two-way ANOVA followed by Sidak multiple comparisons test, n = 4 mice/group. Data are expressed as mean +/− SEM. A set of molecular markers was applied to assess astrocyte activation and polarization towards pro-inflammatory or anti-inflammatory phenotype at 2 weeks post-injury. While *lcn2* was significantly upregulated in xCT−/− spinal cord, no other changes in astrocyte markers were observed between genotypes (**c**,**d**). ***p < 0.001, Mann–Whitney test, n = 3–4 mice/group. Data are expressed as mean + /− SEM. Spinal cords from both genotypes were immunostained with Iba1 antibody and morphology-based-microglia activation was sorted according to four types: from a morphology with long and thin ramified processes type A to an amoeboid morphotype D (**e**). The sorting of Iba1-positive cells was performed in the ipsilateral hemicord at epicenter, and + 500 µm and + 1000 µm caudal to the epicenter (**f**). Two weeks-post-injury, epicenter and perilesional areas (+ 500 µm and + 1000 µm away) of xCT−/− mice contained significantly more “type A” Iba1-positive cells. A set of molecular markers was applied to assess microglia polarization towards pro-inflammatory or anti-inflammatory profile at 2 weeks post-injury. The gene expression of *nox2* and *tnf-a* was lower while *ym1* and *igf-1* were significantly higher in xCT−/− mice (**g**). Data are expressed as mean +/− SEM. *p < 0.05, Mann–Whitney test, n = 3–4 mice/group; ^a^*p* < 0.05, ^a’^*p* = 0.059, Mann–Whitney test for “type A” microglia between xCT+/+ and xCT−/− mice, n = 4 mice/group; ^b^*p* < 0.05, Mann–Whitney test for “type B” microglia between xCT+/+ and xCT−/− mice, n = 4 mice/group. Scale bars = 50 µm in panel A and 20 µm in panel E.

As we previously found an expression of xCT in spinal astrocytes, we wondered how its deficiency would impact on their reactivity following SCI. Anti-GFAP immunohistochemistry was applied onto the injured spinal slides to evaluate the extent of reactive astrogliosis in both groups (Fig. 5a). The number of GFAP + astrocytes in the ipsilateral hemicord was counted at different distances around the epicenter and did not reach the statistical difference (Fig. 5b). We sought to use a more sensitive marker of reactive astrocytosis by quantifying the transcript of lipocalin-2 (*lcn2*) at two weeks post-SCI. *lcn2* mRNA was significantly upregulated in xCT−/− spinal cords compared to wild-type littermates (Mann–Whitney test; *p* = 0.0001) (Fig. 5c). We next used a set of molecular markers to characterize the polarization of reactive astrocytes towards classic proinflammatory *versus* neuroprotective phenotypes. Accordingly, we compared mRNA expression levels of specific astrocyte-associated proinflammatory (*c3, gbp2, ligp1, clcf1*) and neuroprotective (*serping1, emp1, ptgs2*) genes^30^. None of the mRNAs investigated showed a differential expression between injured xCT+/+ and xCT−/− spinal tissues at 2 weeks post-injury (Fig. 5d).

### Injured xCT−/− spinal cords accumulate more ramified microglia/macrophages, bearing an anti-inflammatory phenotype

We first characterized the number of spinal microglia*/macrophages* using anti-Iba1 immunohistochemistry (Fig. 5e). According to morphology-based criteria^31^, thin and highly ramified Iba1 + cells were considered as inactivated/resting “type A” microglia*/macrophages* and hypertrophied amoeboid Iba1 + cells devoid of cell process were considered as fully activated “type D” microglia*/macrophages*; type B and type C being intermediary activated states. Significant changes in cell number or shape were observed at two weeks post-SCI in the injured xCT−/− spinal cords, with an increased number of A microglia*/macrophages* in the lesion epicenter and in regions caudal to the epicenter (Mann–Whitney test, *p* = 0.028, *p* = 0.028 and *p* = 0.028 at epicenter, 500 µm and 1000 µm respectively), together with a decreased number of type B microglia 500 µm away of the epicenter (Mann–Whitney test, *p* = 0.029) (Fig. 5f). At 6 weeks post-SCI, there was also a trend for a higher number of type A microglia*/macrophages* in the xCT−/− spinal cords (Mann–Whitney test, *p* = 0.059). As the deletion of xCT had been shown to influence M1/M2 microglia polarization in the spinal cord of a mouse model with symptomatic ALS^27^, we sought to know how xCT+/+ or xCT−/− microglia*/macrophages* behave following SCI. Using a battery of molecular markers, we pointed out an imbalance in the inflammatory polarization at 2 weeks post-injury. Among the pro-infammatory genes, *nox2* and *tnf-a* were downregulated (Mann–Whitney test, *p* = 0.050 and *p* = 0.027 respectively) while among the anti-inflammatory genes, *ym1* and *igf-1* were upregulated (Mann–Whitney test, p = 0.042 and p = 0.031 respectively) in the injured xCT−/− mice (Fig. 5g).

## Discussion

In the present manuscript, we sought to characterize the cellular distribution of xCT expression in the normal and injured mouse spinal cord, as well as the effect of xCT deficiency on functional and histopathological SCI outcomes. Based on a validation approach using specific *ish* probe and xCT knock-out tissue, we first showed that *slc7a11*/xCT mRNA was significantly detected in the normal mouse spinal cord and was preferentially distributed in meningeal cells, vascular mural cells, astrocytes, microglia and motor neurons. As demonstrated by the double labeling, *slc7a11*/xCT mRNA was not found in the satellite oligodendrocytes. Our results, although based on in situ gene expression, are partly in line with those showing xCT protein in meningeal cells^32,33^, in astrocyte subpopulations^23,34,35^ and in microglia^17–19,27,32,35–37^, while questioning an expression by oligodendrocytes^20–22,34^ or neuron subtypes described elsewhere^19,22,37^. Instead, *slc7a11*/xCT *ish* signal was widely distributed in gray matter neuropil where numerous neuronal and glial processes interwove, with a high density throughout Rexed laminae I to III. The reason for such an enrichment is unknown but one can hypothesize that a glial glutamate transporter, in close proximity to sensory neurons, could locally modulate the glutamatergic neurotransmission and the sensory processing. Recent data on xCT protein localization in the adult mouse spinal cord support our *ish* findings^38^. After injury, the cell types expressing xCT mRNA were unchanged, taking into consideration the limited set of molecular markers herein investigated.

Following SCI, *slc7a11*/xCT gene expression was upregulated, as early as the fourth day post-injury. This high expression was not statistically significant at later timepoints but, still plateaued for a duration lasting up to 6 weeks post-injury. Using a commercial anti-xCT antibody, whose specificity was validated on knock-out CNS homogenates (brain and spinal cord), we confirmed that xCT protein level significantly increased during the sub-acute phase of experimental SCI. The sub-acute phase—from first hours to two weeks post-SCI—is characterized by an overall dysregulation of ions and neurotransmitters, blood–brain barrier leakages, vascular remodeling, local energy shortage, production of inflammatory mediators and free radicals from recruited immune cells or resident activated glial cells. Altogether, these changes disrupt the spinal homeostasis one way or another, and contribute to the secondary neuronal damage. Given the dual role of xCT in CNS tissue homeostasis and its early overexpression following SCI, we wondered how the lesion extension, nerve cell survival, glial activation or neuro-inflammation would be impacted in xCT knock-out mice. On one hand, the consequence of System xc− deficiency could be deleterious by reducing the import of cystine and affecting the anti-oxidant capacity; on the other hand, it could decrease the threshold for excitotoxicity and thus protect surrounding spinal tissue.

In this study, we showed that spinally-injured xCT knock-out mice had a faster weight and grip strength recovery than wild-type littermates, especially during the first two weeks post-injury, which suggests an early involvement of xCT on functional recovery. Albeit the rostro-caudal extension and volume lesion were similar in both genotypes after injury, the analysis of spinal tissues demonstrated that motor neuron loss and activated microglia, as well as the levels of pro-inflammatory markers, were less pronounced in injured xCT−/− mice. As xCT mRNA is expressed by both non-neuronal cells and motor neurons, the impact of its absence on neuron survival can be explained by either a cell autonomous or a non-cell autonomous protective effect. Supporting the influence of non-neuronal System xc−, an in vitro study demonstrated that neuronal killing by macrophage-mediated glutamate release was dependent on System xc− activity^39^. Another in vitro model using neuron-astrocyte co-culture showed that, during hypoglycemic stress, glutamate released from astrocytes via System xc− is largely responsible for excitotoxic neuron death^40,41^. Specifically, in the spinal cord, it has been shown that the local activation of System xc−, upon intraspinal LPS plus cystine administration, exerts neurotoxicity towards (motor) neurons^36^. Glutamate excitotoxicity is classically described as one of the precipitating events leading to secondary motor neuron death after SCI^1–3,42^. Abnormal extracellular glutamate levels are common at the lesion epicenter due to uncontrolled release during neuronal death but also due to the chronic downregulation of astrocytic EAATs^1–3,42^. Increased expression or activity of System xc− by activated glial cells at the SCI epicenter could add to the unwanted extracellular glutamate accumulation. Measuring System xc− activity in the spinal cord is technically challenging and would have implied for instance in vivo microdialysis or specific radiolabeled ligands^43^. Although very informative, we did not implement these approaches in the present SCI paradigm. Instead, we quantified the intraspinal levels of glutathione pre- and post-SCI. The intraspinal levels of the reduced form of glutathione were equally lowered in both genotypes after injury, likewise the levels of carbonylated proteins, common markers of oxidative stress^44^. Overall, this suggests that xCT deficiency in injured knock-out mice does not deleteriously alter the levels of anti-oxidant countermeasures.

Surprisingly, the loss of oligodendrocytes was similar in both spinally-injured genotypes whereas we could have expected a difference in cell survival if glutamate excitotoxicity was suspected as the mechanism of System xc- neurotoxicity. Indeed, just like motor neurons, oligodendrocytes express significant levels of calcium-permeable ionotropic glutamate receptors, making them susceptible to excitotoxic cell death. The main limitation in the interpretation of this result is that the p25a marker labels mature oligodendrocytes as well as oligodendrocyte precursor cells, known to highly proliferate as early as 1-week post-injury^45^. Therefore, we may have not caught the detailed picture of oligodendrocyte loss looking at two weeks post-injury. In the future, this should be taken into consideration as several reports also point out a non-cell autonomous effect of System xc- on oligodendrocyte survival in models of demyelinating diseases^18,28^.

In a number of experimental models of CNS disorders, xCT inhibition, using either genetic or pharmacological approaches, has been found to be more often neuroprotective than deleterious e.g. in 6OHDA-induced Parkinson’s disease^12^ and middle cerebral artery occlusion^15^. With respect to spinal cord diseases, xCT deletion in SOD1^G37R^ mice slows down ALS disease course by mitigating spinal motor neuron loss and microglia activation^27^. In mouse models of experimental autoimmune encephalomyelitis, mutant Slc7a11^sut/sut^ mice and mice treated with xCT inhibitors sulfasalazine or S-4-carboxyphenylglycine showed both better clinical outcomes, associated with enhanced oligodendrocyte survival and reduced immune cell infiltration^28^. These data strengthen the idea that xCT upregulation or enhanced activity during neurologic conditions compromises spinal tissue integrity and neuron/oligodendrocyte survival. The mechanism underlying the neuroprotection in the cases of ALS or EAE experimental models combined with xCT deficiency, clearly involved an anti-inflammatory polarization of the immune response. In an attempt to extend Evonuk’s findings, Merckx and colleagues induced EAE in xCT full knock-out mice but did not observe any difference in the clinical outcomes between wild-type and xCT-deficient mice^29^. However, in chimeric animals expressing xCT in their entire body except for their bone marrow-derived cells -and thus immune cells- the authors found better clinical scores compared to wild-type mice after EAE induction. This suggests that System xc− modulates macrophage/microglia function, most likely via changes in glutamate release or intracellular redox balance^27,36^.

Several lines of evidence now support that System xc− regulates the way microglia and macrophages polarize or activate along their inflammatory response^27,35^. In our model of SCI, we showed an impact of xCT deletion on the degree of microglia/macrophage activation; within the first two weeks post-SCI, injured xCT−/− spinal cords had an increased number of resting “type A” microglia/macrophage at several distances around the epicenter. It would have been interesting to decipher from microglia or macrophages which cells were involved in such difference, by using for instance specific TMEM119 microglia marker. Overall, it means that the activation of microglia or macrophages is alleviated on the full xCT knock-out background. It is classically described that equal amount of M1/pro-inflammatory and M2/anti-inflammatory macrophages/microglia invade the SCI lesion within the first week^46–49^. At the end of second week post-SCI, M1 macrophages usually outnumbered M2 cells^46^. According to our gene expression profile, the cellular response within the injured spinal tissue was early shifted towards an anti-inflammatory phenotype in xCT-deficient mice. In particular, *nox2* and *tnf-a* mRNA were significantly lowered while *ym1* and *igf-1* mRNA levels were upregulated. These findings on pro-to-anti-inflamatory gene response have been described in a similar context when xCT−/− mice were crossed with mutant SOD1^G37R^ ALS mice and ALS disease course was slowed down^27^. Also, xCT-deficiency alleviates the release of pro-inflammatory cytokines in the cerebral cortex or hippocampus in a model of LPS-induced post-septic neurological psychiatric illness^23,35^.

Finally, we sought to decipher whether xCT-deficiency would have an impact on the polarization of the astrocyte response during the sub-acute phase of SCI. Although the number of GFAP + astrocytes did not differ, xCT-deficiency might modulate astrocyte reactivity following nervous trauma. All astrocyte-derived inflammatory markers were similarly modulated in both groups. However, lipocalin-2, that is mostly expressed by astrocytes under inflammatory conditions^50,51^, was induced in injured xCT−/− spinal cords. In models of LPS-induced sepsis or retinal degeneration, recent reports have shown that lipocalin-2 can serve as a potent neuroprotective factor in response to neuro-inflammation^52–54^. Surprisingly, knocking-out lipocalin-2 in mice undergoing SCI improves functional and histopathological outcomes compared to wild-type mice^55^. The contribution of lipocalin-2 in diseases characterized with prominent neuro-inflammation or astrocyte activation is thus still confusing.

The pathological events following SCI involve many cellular and molecular actors that operate in a time-regulated fashion, so that the only way to aim at deciphering whether System xc− from astrocyte and/or microglia modulate neuro-inflammation pathways would be to investigate inducible conditional knock-out mice models. So far, our results show that full genetic xCT invalidation modifies functional and histopathological SCI outcomes. In the future, pharmacological inhibition of System xc-, using BBB-permeant specific inhibitors, might be a therapeutic approach in order to accelerate post-SCI recovery in patients.

## Methods

### Animals

All aspects (ethical, data collection, data analysis) included in this study are reported in accordance with ARRIVE guidelines. The experimental protocol was conducted in compliance with the European Communities Council Directives for Animal Experiments (2010/63/EU, 86/609/EEC and 87/848/EEC) and was approved by the Animal Ethics Committee of the University of Namur (ethic project UN 17-295). Male C57BL/6J mice, aged from 3 to 4 months were included in the study. xCT−/− mice and their xCT+/+ littermates have a C57BL/6J background and are high-generation descendants of the strain described previously^56^. Genotypes were confirmed by PCR amplification of ear DNA using REDExtract-N-Amp Tissue PCR Kit (Sigma-Aldrich, Overijse, BE) and the following primers: 5′-GATGCCCTTCAGCTCGATGCGGTTCACCAG-3′ (GFPR3); 5′-CAGAGCAGCCCTAAGGCACTTTCC-3′ [mxCT5′flankF6]; 5′-CCGATGACGCTGCCGATGATGATGG-3′ [mxCT(Dr4)R8].

### Spinal cord injury

After genotyping, xCT−/− mice and xCT+/+ littermates were randomly subjected to a spinal cord contusion. Mice were anesthetized with a cocktail of ketamine (100 mg/kg) and xylazine (5 mg/kg). The full SCI procedure was similar as described previously, except that the spinal impact targeted C5-only^57^. Briefly, after incision of the dorsal skin and underlying muscles between C2 and T1 bony processes, the paravertebral muscles overlying C4–C6 were removed. A unilateral laminectomy on the right side of C5, extending on top of the posterior spinal artery, was performed followed by the stabilization of the spinous processes of C2 and T2 using toothed Adson forceps^57^. xCT+/+ and xCT−/− mice were subjected to spinal contusion using the computer-controlled Infinite Horizon IH400 impactor (Precision Systems and Instrumentation, Lexington, USA). The C5 impact was generated with a force set at 50 kD. The impact force sensed by the IH device was 52.3 ± 0.3 kD (mean ± SEM) for all injuries. The parameters (i.e., displacement and velocity of the tip) are listed per genotype in Table 1. None of the parameters were significantly different between xCT+/+ and xCT−/− mice. At the end of the surgery, overlying muscles were closed in layers with sterile 4–0 silk suture, and the skin incision was closed using sterile wound clips. Animals were allowed to recover on a circulating warm water heating pad until awake and then returned to their home cages. Animals were monitored on a daily basis for 5 days, and measures were taken to avoid dehydration and to minimize any potential pain (subcutaneous administration of buprenorphine: 0.1 mg/kg).

**Table 1:** Parameters recorded after computer-controlled SCI contusion.

	xCT+/+	xCT −/−	Significance
Force (kD)	51.9 ± 0.4	52.7 ± 0.5	*p* > 0.05
Velocity (mm/sec)	123.0 ± 0.6	122.8 ± 0.4	*p* > 0.05
Displacement (µm)	669.8 ± 21.3	713.2 ± 19.9	*p* > 0.05

Data are expressed as mean ± SEM.

### Cylinder paw preference test

All behavioral measurements were conducted on mice starting 2 weeks before injury and thereafter on a weekly basis until they were sacrificed. Mice were weekly video-recorded while rearing freely in a transparent cylinder for 5 min^58^. This test uses spontaneous rearing and exploratory behavior to evaluate forelimb asymmetries under a preference paradigm. Three behaviors were scored during vertical exploration: use of the right ipsilateral forelimb alone, use of the left contralateral forelimb alone, and use of both forelimbs simultaneously. The hit score was expressed as a percentage of usage of each forelimb to total limb usage. The video analysis was performed by one investigator (L.S.) who was blinded to the genotypes.

### Grip strength test

Muscle grip strength was determined weekly for the ipsilateral (right) forelimb using a “grip strength meter” Model GS3 (Bioseb, Vitrolles, France). Grip strength testing was performed by allowing the mouse to grasp a thin bar attached to the force gauge^58^. This was followed by pulling the animal away from the gauge until the forelimb released the bar. The force measurements were averaged from three trials. The test was performed by one investigator (C.N.) who was blinded to the genotypes.

### In situ hybridization, histology and immunohistochemistry

*Tissue processing.* Under anesthesia (ketamine (100 mg/kg) and xylazine (5 mg/kg)), mice were exsanguinated and transcardially perfused with NaCl 0.9% followed by phosphate-buffered 4% paraformaldehyde. Spinal cords were harvested and post-fixed overnight in fixative. Cervical spinal regions were then dehydrated, paraffin-embedded and cut into 10-µm serial sections.

in situ hybridization *(ish).* Chromogenic *ish* detection was performed according to the manufacturer’s protocol (RNAScope 2.5 HD Detection Reagent RED, #322360, Advanced Cell Diagnostics Inc, Hayward, USA). Paraffin-embedded spinal cord sections were incubated at 60 °C for 1 h followed by deparaffinization and dehydration. Endogenous peroxidases were blocked with hydrogen peroxide for 10 min at room temperature (RT) followed by 2 washes in milliQ water. Heat-induced epitope retrieval was performed for 15 min at 100 °C using RNAScope Target retrieval followed by 2 milliQ washes and a 99% ethanol rinse. Slides were dried at RT and hydrophobic barriers were applied. Samples were then incubated with RNAScope protease plus at 40 °C for 30 min and subsequently washed in milliQ water. Samples were incubated with the probe specifically targeting *slc7a11* mRNA (RNAScope ref. 422511, Advanced Cell Diagnostics Inc, Hayward, USA) for 2 h at 40 °C and then washed for 2 min at RT in RNAScope wash buffer. Amplification rounds 1–6 alternated from 30 to 15 min at 40 °C. After each amplification round, samples were washed with RNAScope wash buffer. Signal detection was performed by incubating samples with alkaline phosphatase (solution of RNAScope Fast B and A ratio 1:60) for 10 min at RT. Samples were then washed in milliQ water. Following *ish*, samples were either counterstained with hematoxylin (Sigma, St Louis, MO), dried for 30 min at 60 °C and mounted using VectaMount (Vector Laboratories, Burlingame, USA) or fluorescently stained following the protocol below.

For fluorescent *ish*/immunofluorescence multiplexing, detection was performed according to the manufacturer’s protocol (RNAScope Multiplex Fluorescent Detection Reagent V2, #323110, Advanced Cell Diagnostics Inc, Hayward, USA). Briefly, after *slc7a11* probe incubation of 2 h at 40 °C (RNAScope ref. 422511-C2, Advanced Cell Diagnostics Inc, Hayward, USA), sections were washed with RNAScope wash buffer. Three amplification steps were performed and 15 min of HRP C2 incubation at 40 °C was necessary. After 3 × 5 min washes, tissue sections were incubated with TSA Plus fluorophore 1:1500 for 30 min at 40 °C. After 15 min incubation of HRP blocker at 40 °C, sections were washed in Tris-buffered saline supplemented with 0,1% Tween (TBS-T). Endogenous peroxidases were blocked with a mix of horse and goat serum (Dako, Glostrup, Denmark) for 30 min at RT. After 3 × 5 min washes, samples were incubated with primary antibodies overnight at RT (Table 2). After that, samples were washed with TBS-T. Fluorophore-coupled secondary antibodies alexa fluor 488 anti-mouse or anti-rabbit 1:100 (Jackson ImmunoResearch Inc, West Grove, PA) were applied on their respective tissue sections and incubated for 30 min at RT. Afterwards, they were washed with TBS-T. Finally, tissue sections were coverslipped with ProLong Gold mounting medium containing DAPI (ThermoFisher Scientific, USA).

**Table 2:** List of antibodies.

Antigen	Supplier - reference	Multiplex IF dilution	IHC dilution
GFAP	Sigma-Aldrich clone GA5–G3893	1 :40	1 :1000
Iba1	Wako Chemicals 019-19741	1 :100	1 :300
MAP2	Genetex 1D7-B9-C8-F6	1 :100	n.a
p25α	Sigma-Aldrich HPA-036576	1 :100	1 :300
Vimentin	Abcam Ab92547	1 :500	n.a

*Lesion histology and motor neuron counting.* Using every fifth slide, paraffin sections were dewaxed, rehydrated and stained with Eriochrome Cyanine R/Neutral red. Sections were dehydrated, mounted in DPX (Merck Millipore, Darmstadt, DE) and imaged using Olympus BX63 upright microscope. Using ImageJ software, the lesion area was outlined and quantified from caudal to rostral side around the epicenter. Specifically, the lesion area was determined every 150 μm on Neutral Red sections and expressed as a percentage of total area of the hemi-spinal cord ipsilateral to the lesion. Injury included areas of both lost tissue and surrounding damaged tissue where the normal microanatomical structure of the spinal cord was disrupted. The lesion epicenter was considered as the section with the largest percent of injured tissue. As previously described, the lesion volume was determined using the Cavalieri estimator of volume equation: V = [Σ (A1 + A2 + ··· + An) × D] − [Amax × Y] with A = damaged surface (μm^2^), D = distance between 2 Sects. (150 μm) and Y = section thickness (10 μm)^57^.

In Neutral Red stained sections, the total number of cervical motor neurons was quantified in a blind manner. Ventral horn was defined as gray matter ventral to the central canal. Only motor neurons with a clearly identifiable nucleus and a cell soma > 150 μm^2^ were counted.

*Immunohistochemistry and glial cell counting.* Oligodendrocytes, astrocytes and microglia were revealed respectively using anti-p25α, anti-GFAP and anti-Iba1 immunolabelings. Using every fifth slide, paraffin sections were dewaxed, rehydrated and subjected to heat-induced epitope retrieval in citrate buffer pH 6 at 100 °C for 10 min. Endogenous peroxidase activity was abrogated with 3% H_2_O_2_ in methanol for 10 min. To avoid non-specific binding, a bath of glycine 0,1 M was performed for 3 min followed by a blocking step in 5% goat or horse serum diluted in TBS for 30 min. Sections were then incubated overnight at 4 °C with primary antibodies diluted in TBS with 1% normal serum (Table 2). The next day, secondary biotinylated antibodies (1:300; Vectastain, ABC Kit, USA) were incubated on tissue slices 1 h at room temperature. Sections were then incubated with a solution of peroxidase-bound streptavidin (1:200-Vectastain) for 45 min. Immunoreactivity was revealed using 3,3 di-amino-benzidine (Dako, Glostrup, DK). Finally, sections were counterstained with hemalum, dehydrated and mounted in DPX. Each slice was imaged with an Olympus BX63 microscope using the Cell Sens software, and the number of immunolabelled cells was counted in the ipsilateral hemicord then plotted rostrally and caudally around the lesion epicenter.

### Image quantification

Except for neuron counting, the quantification of cell densities was performed in the lesion epicenter (0 µm) and two regions caudal to it (respectively at +500 and +1000 µm distances). The epicenter was set as abovementioned. One slice per region was acquired for each animal and imported into ImageJ software (https://imagej.nih.gov/ij/). The quantitative evaluation of immunostainings was blindly performed by one investigator (L.S.): total immunoreactive cells (GFAP+ , p25a+ or Iba1+) were manually counted within the whole ipsilateral hemicord. The morphology of Iba1+ microglial cells was analyzed according to a previously described protocol^31^. In each hemicord slice, all Iba1+ cells were counted and classified into four types based on their morphology. Type A resting: cells have long and very thin processes; Type B ramified: processes are long and dense, cytoplasm around nucleus is visible; Type C ramified: processes are shorter and there are many branches; Type D amoeboid: there is no more processes, the cell is limited to nucleus and strong immunoreactive cytoplasm.

### Western blot and OxyBlot

Proteins were extracted from 2-mm pieces of spinal cord centered on C5 level. Tissue was crushed using a Tissue Grinder in 200 µl of RIPA lysis buffer for 2 h on ice. After centrifugation at 13,000×g for 5 min at 4 °C, the quantity of proteins in supernatant was measured with the Pierce protein assay kit (Thermo Scientific, Bleiswijk, NL). Samples were mixed with 100 nM DTT and 5% β-mercaptoethanol, and incubated 1 h at 37 °C prior to gel loading. Proteins (20 µg) were loaded on a 12% polyacrylamide gel, separated via SDS-PAGE and transferred to a PVDF membrane (120 V for 1h30). Membranes were incubated with a blocking solution containing 5% milk in TBS-Tween for 30 min, followed by incubation with primary antibodies diluted in 5% milk in TBS-Tween at 4 °C overnight. Primary antibodies used were rabbit anti-xCT antibody (Cell Signaling Technology #98051S, 1:500), mouse anti-GAPDH (Sigma-Aldrich G8795, 1:10,000) or mouse anti-β-actin antibody (Sigma-Aldrich A5441, 1:10,000) diluted in 5% TBS-BSA. Specificity of xCT antibody was carefully investigated using negative and positive tissue controls (Supplementary Fig. 1). Detection of protein carbonyls formation was performed using an OxyBlot kit according to the manufacturer’s instructions (cat #S7150; Merck Millipore). Membranes were then rinsed and incubated with an anti-rabbit or an anti-mouse HRP-linked antibody (Cell Signaling #7074S or #7076S) diluted 1:1000 in 5% milk in TBS-Tween. Signal was revealed using a chemiluminescent method (BM chemiluminescence blotting substrate (POD), Roche Diagnostics, Mannheim, DE) and imaged on an Image Quant LAS 4000 mini using LAS 4000 luminescent image analyzer (GE Healthcare, Diegem, BE). The number of pixels for each band-of-interest was quantified after importation in ImageJ and expressed as related to the respective loading control (GAPDH).

### Glutathione assay

Two-mm pieces of spinal cord centered on C5 level were homogenized in ice-cold PBS. Homogenates were centrifuged for 15 min at 10,000×g and the protein content of the supernatant was determined using the Pierce protein assay kit (Thermo Scientific, Bleiswijk, NL). The GSH content of the supernatant was analyzed using a QuantiChrom GSH Assay Kit according to the instructions of the manufacturer (BioAssay Systems, Hayward, USA).

### RNA isolation and qRT-PCR

Two-mm pieces of spinal cord centered on C5 level were homogenized in Trizol reagent (Life Technologies, Bleiswijk, NL). Total RNA was further isolated according to the manufacturer’s instructions including a step of purification using the High Pure RNA Tissue Kit (Roche Diagnostics, Basel, CH). RNA concentrations were measured using a spectrophotometer Nanodrop 1000 (Thermo Scientific, Bleiswijk, NL). One µg of total RNA was reverse-transcribed using the High Capacity cDNA Reverse Transcription Kit according to the manufacturer’s instructions, including DNAse treatment (Applied Biosystems, Thermo Fisher Scientific Baltics, LT). One µL of cDNA was amplified using specific primers targeting genes-of-interest with the Takyon SYBR Green according to the manufacturer’s instructions (Eurogentec, Liège, BE) in the Light Cycler 96 system (Roche Diagnostics, Mannheim, DE). Primer sequences are listed in Table 3. Specific amplification was confirmed by melting curve analysis. Relative gene expression was computed using the 2-ddCq method with HPRT as housekeeping gene.

**Table 3:** List of primers.

Gene accession number	Sequence	Amplicon size
*arg1*	Sense 5′-GTACATTGGCTTGCGAGACG-3′	209 bp
NM_007482.3	Antisense 5′-TTTCTTCCTTCCCAGCAGGT-3′	
*c3*	Sense 5′-CTGGCCCTGATGAACAAACT-3′	116 bp
NM_009778.3	Antisense 5′-GGATGTGGCCTCTACGTTGT-3′	
*ccl2*	Sense 5′-AGGTCCCTGTCATGCTTCTG-3′	136 bp
NM_011333.3	Antisense 5′-CGTTAACTGCATCTGGCTGA-3′	
*clcf1*	Sense 5′-CTGCCACTTGCCAGTACTCA-3′	85 bp
NM_001310038.2	Antisense 5′-CCTTATCCCAGAAAGGCACA-3′	
*emp1*	Sense 5′-GATGCTATCAAGGCAGTGCA-3′	125 bp
NM_001288627.1	Antisense 5′-GGACCCTGAGAGGAAGAACC-3′	
*gbp2*	Sense 5′-AAGAGCCTGGTGCAGACCTA-3′	107 bp
NM_010260.1	Antisense 5′-TTGCACTGCTGCTGAGTTCT-3′	
*hprt*	Sense 5′-GGACCTCTCGAAGTGTTGGAT-3′	186 bp
NM_013556.2	Antisense 5′-CCAACAACAAACTTGTCT-3′	
*igf-1*	Sense 5′-TACCCTACATGTGCATTTGCA-3′	185 bp
NM_001314010.1	Antisense 5′-CACAGACATGCCACATTTCAC-3′	
*il-1b*	Sense 5′-CCCAAGCAATACCCAAAGAA-3′	230 bp
NM_008361.4	Antisense 5′-GCTTGTGCTCTGCTTGTGAG-3′	
*lcn2*	Sense 5′-CTG-AAT-GGG-TGG-TGA-GTG-TG-3′	100 bp
NM_008491.1	Antisense 5′-GCT-CTC-TGG-CAA-CAG-GAA-AG-3′	
*ligp1*	Sense 5′-AATACCTGCCTCACGCTCAT-3′	107 bp
NM_001146275.1	Antisense 5′-TTCTTAACCACTGGGCCAAC-3′	
*nos2*	Sense 5′-CAATGGCAACATCAGGTCGG-3′	170 bp
NM_001313921.1	Antisense 5′-CGTACCGGATGAGCTGTGAA-3′	
*nox2*	Sense 5′-ACTGCGGAGAGTTTGGAAGA-3′	201 bp
NM_007807.5	Antisense 5′-GGTGATGACCACCTTTTGCT-3′	
*ptgs2*	Sense 5′-TCCTCCTGGAACATGGACTC-3′	108 bp
NM_011198.4	Antisense 5′-TTTGCCACTGCTTGTACAGC-3′	
*serping1*	Sense 5′-GGTTGAGACAGGCTTGGGTA-3′	103 bp
NM_009776.3	Antisense 5′-CTGCCAGTTCCTAAGGCTTG-3′	
*slc7a11*	Sense 5′-GAC-GAT-GGT-GAT-GCT-CTT-CTC-3′	118 bp
NM_011990.2	Antisense 5′-TGG-GCG-TTT-GTA-TCG-AAG-ATA-3′	
*tgf-b*	Sense 5′-TTGCTTCAGCTCCACAGAGA-3′	183 bp
NM_011577.2	Antisense 5′-TGGTTGTAGAGGGCAAGGAC-3′	
*tnf-a*	Sense 5′-ACGGCATGGATCTCAAAGAC-3′	212 bp
NM_013693.3	Antisense 5′-GTGCGTGAGGAGCACGTAGT-3′	
*ym1/chil3*	Sense 5′-CATGAGCAAGACTTGCGTGAC-3′	173 bp
NM_009892.3	Antisense 5′-GGTCCAAACTTCCATCCTCCA-3′	

### Statistical analysis

Unless specified, all results were expressed as mean values ± Standard Error of Mean (SEM). The data were statistically analyzed using an unpaired T-test when data were spread according to a Gaussian distribution or using a non-parametric Mann–Whitney test in the other cases. For multiple comparisons, one-way (Kruskal–Wallis) or two-way ANOVA tests were also used depending on the number of variables analyzed. The level of significance was set at *p* < 0.05. The statistical analyses were performed by using the software GraphPad Prism version 7 (GraphPad Software, La Jolla, USA).

## Supplementary Information

Supplementary Information.

## Abbreviations

xCT: Specific subunit of the cystine–glutamate exchanger
slc7a11: Solute carrier family 7 member 11
SCI: Spinal cord injury
ALS: Amyotrophic lateral sclerosis
MS: Multiple sclerosis
EAE: Experimental autoimmune encephalitis
GSH: Glutathione
EAAT: Excitatory amino acid transporter
ish: In situ hybridization
