# Supplementary material for: Cystine–glutamate antiporter deletion accelerates motor recovery and improves histological outcomes following spinal cord injury in mice
Source: Sci Rep. 2021 Jun 9;11:12227. doi: 10.1038/s41598-021-91698-y (PMC8190126; doi:10.1038/s41598-021-91698-y)
Supplement: Supplementary file 1 — Supplementary Information. [file 41598_2021_91698_MOESM1_ESM.docx]

**Cystine–glutamate antiporter deletion accelerates motor recovery and improves histological outcomes following spinal cord injury in mice**

Lindsay Sprimont^1^, Pauline Janssen^2^, Kathleen De Swert^1^, Mathias Van Bulck^3^, Ilse Rooman^3^, Jacques Gilloteaux^1,4^, Ann Massie^2^, Charles Nicaise^1^

**Supplementary Figure 1. *In situ* hybridization with a probe targeting *slc7a11* mRNA in spinal cords from xCT+/+ and xCT-/- mice.** The specific *ish* signal from *slc7a11* probe was revealed as pink deposits onto the spinal tissue. Location of *slc7a11 probe* staining across one representative C5 spinal cord section, onto which Rexed laminae were outlined according to the Paxinos Atlas of the Spinal Cord (a). xCT-/- spinal cord was used as tissue control for probe specificity (b). Mice from the breeding colony were genotyped using specific primers for *slc7a11* gene in order to identify the bands corresponding to wild-type +/+ (950 bp), homozygous -/- (1500 bp) or heterozygous +/- (950 bp plus 1500 bp) genotypes (c). Quantitative PCR targeting *slc7a11* mRNA confirmed the absence of gene expression in the spinal cord of xCT-/- mice (same Cq as for water). Since it has been documented that many commercially-available antibodies display non-specific labeling when applied to detect xCT protein in mouse cell lines or tissues, the antibody specificity was verified on xCT-/- and xCT+/+ nervous tissue. Full length blot of xCT detection is displayed where xCT protein was detected at around 43 kDa in mouse spinal cord and brain, with an unexpected consistent band at 130 kDa (d). All these bands were absent in xCT-/- tissues. GAPDH (36 kDa) was used as loading control.

**Supplementary Figure 2. Original membranes from xCT, GAPDH or beta-actin detections.** xCT and GAPDH immunoblots shown in Figure 1d (a-b). Lines 1-4: uninjured spinal cords; lines 5-8: 1-week post-SCI; lines 9-13: 2-week post-SCI. Beta-actin immunoblot shown in Figure 3b (c), used as loading control for Oxyblot. Lines 1-5: xCT+/+; lines 6-10: xCT-/-. Image of these membranes have been acquired on ImageQuant LAS 4000 mini (GE healthcare) with ImageQuant LAS Version 1.3 software. Increments of 30 seconds were applied as exposure type. The presented captures correspond to 2.5 minutes of exposure time. For visibility purposes, the contrast of the blots was enhanced using the automated software interface for xCT and GAPDH immunoblots (a-b). Shorter exposure times have not been recorded given the non-visibility of bands weakly expressed. Membranes borders are highlighted with black solid lines.

**Supplementary Figure 3. Original membranes from xCT and GAPDH detections.**

xCT shown in Suppl. Figure 1d (d1-16) and GAPDH shown in Suppl. Figure 1d (e1-8) immunoblots. Line 1: molecular weight; lines 2-4-6-8: xCT+/+ tissues; lines 3-5-7-9: xCT-/- tissues. Increments of 30 seconds were applied as exposure type. Each picture represents different exposition times from 30 to 480 seconds for xCT (d1-16) and from 30 to 300 seconds for GAPDH (e1-8). The edges of the blots are outlined with black solid lines.


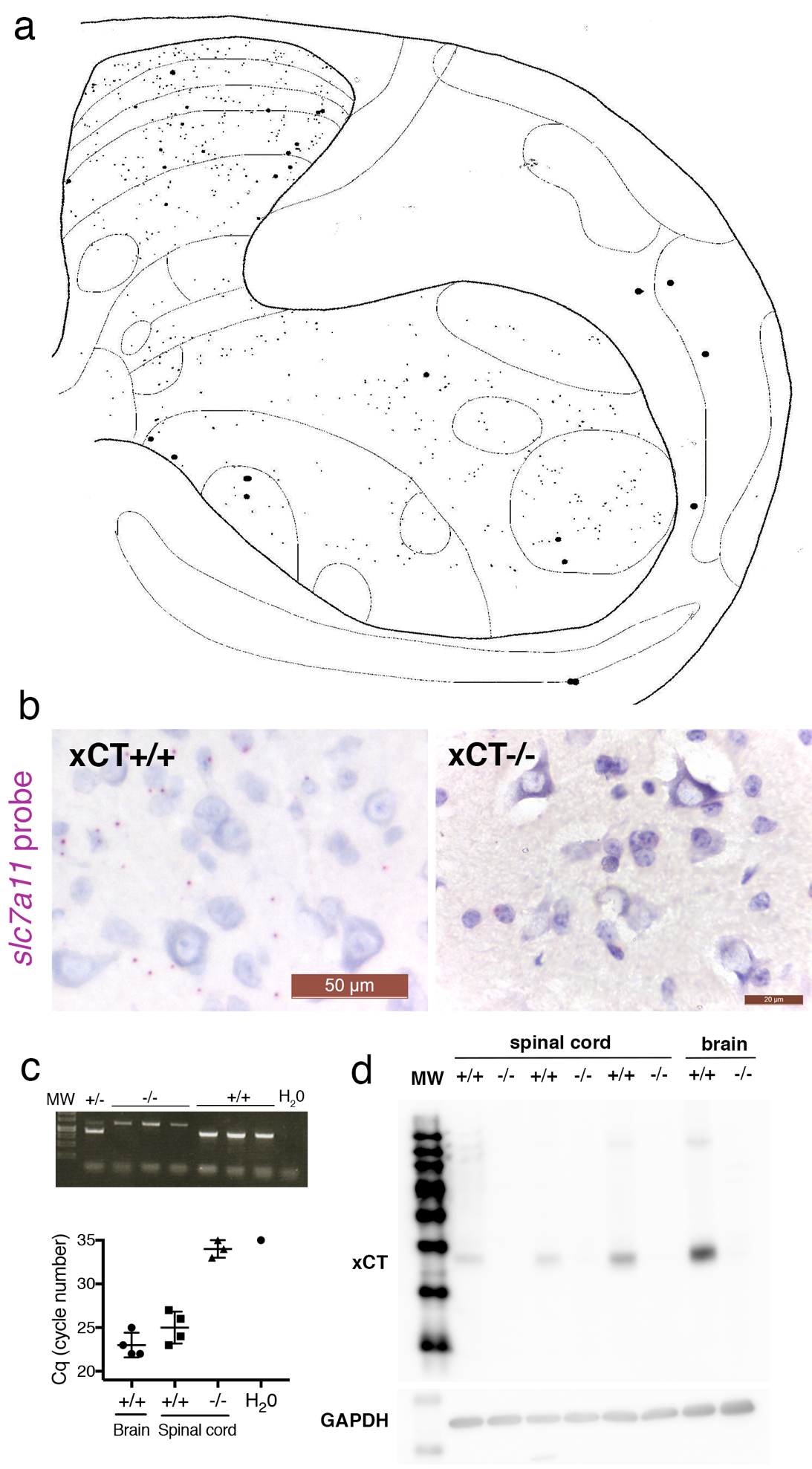


**Suppl. Figure 1.**





a


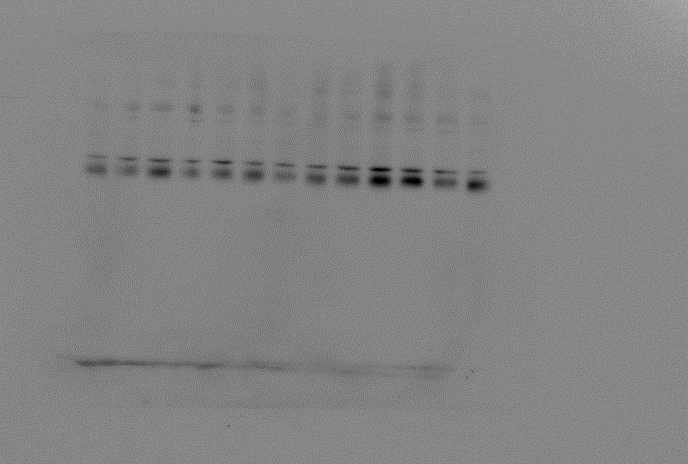


b

c


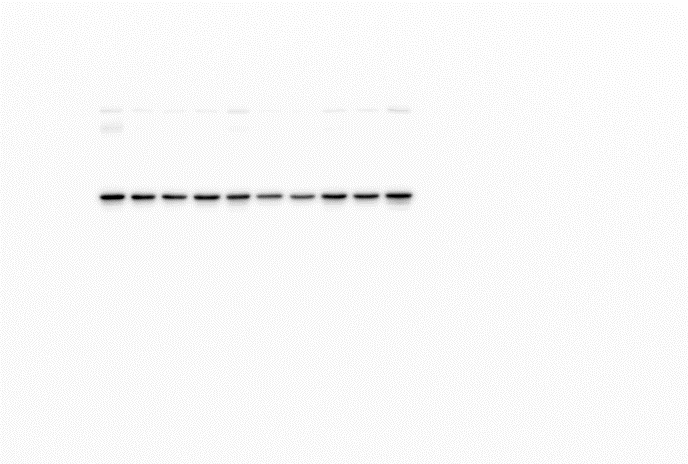


**Suppl. Figure 2.**

d (1-8)


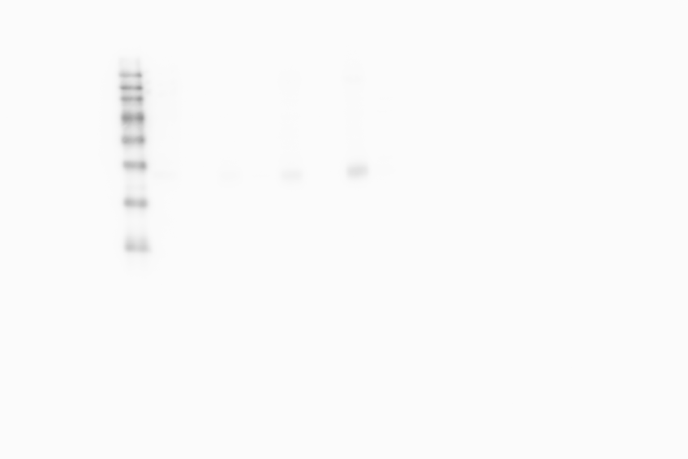

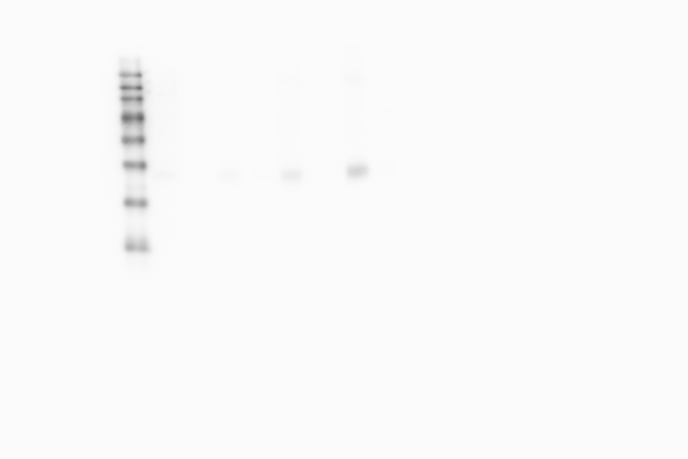

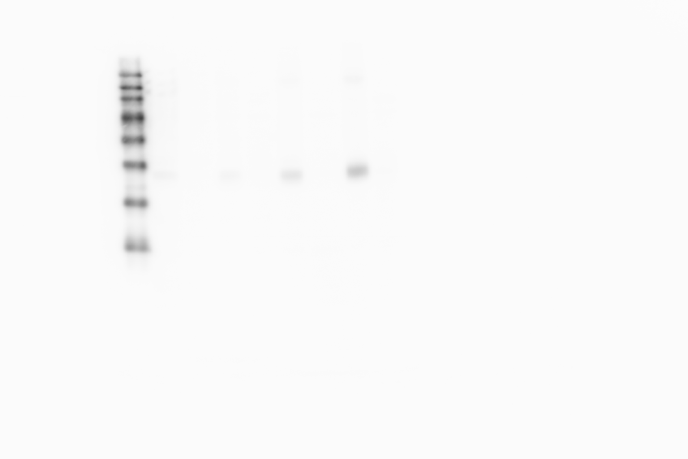

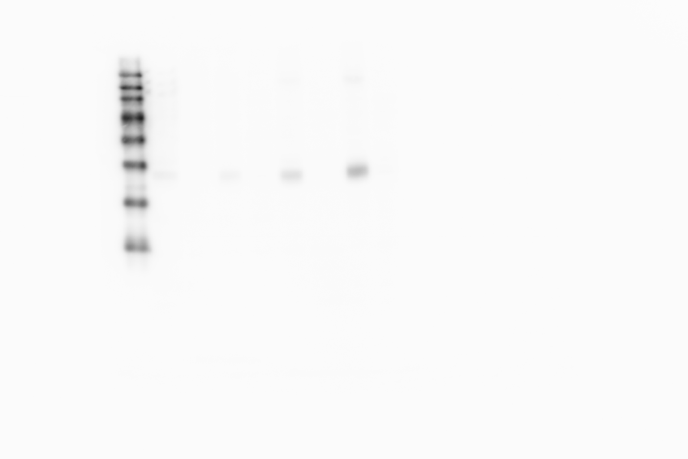

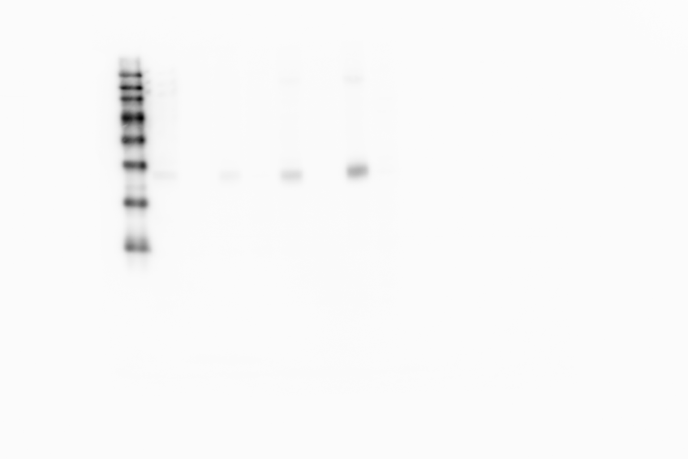

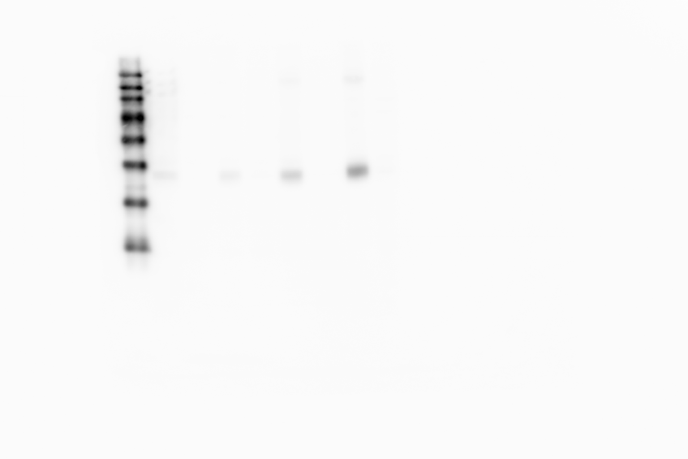

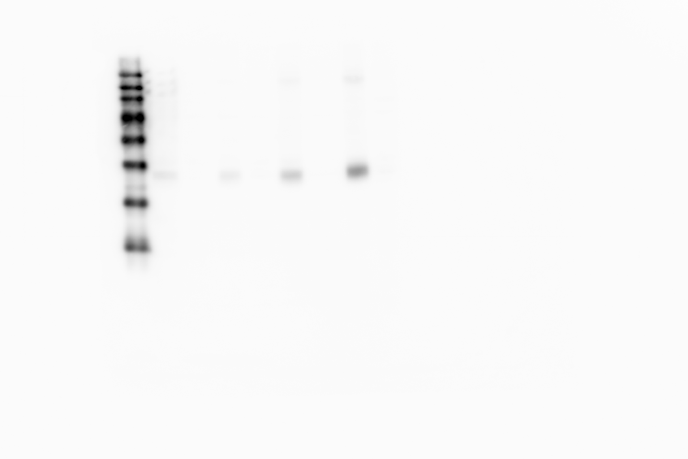

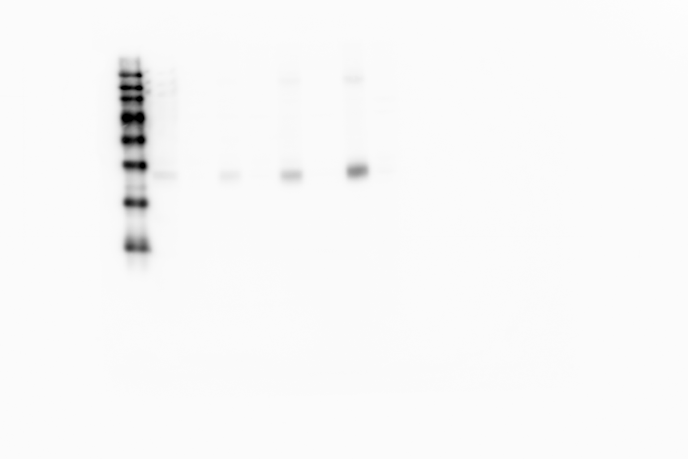

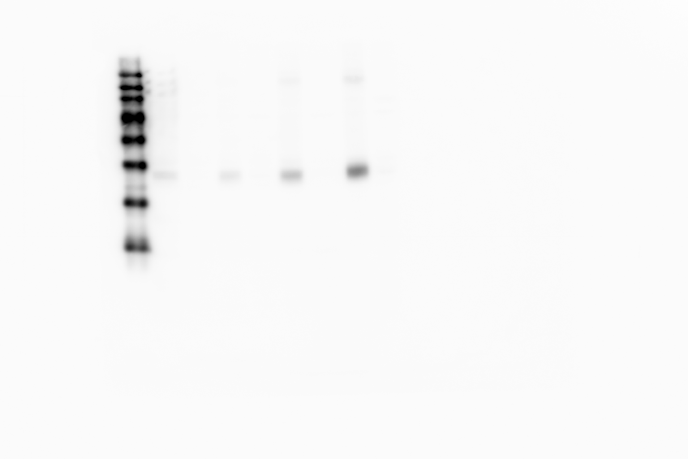

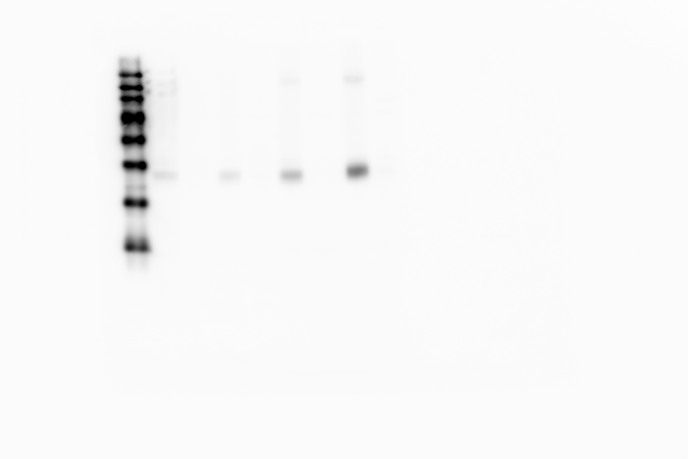

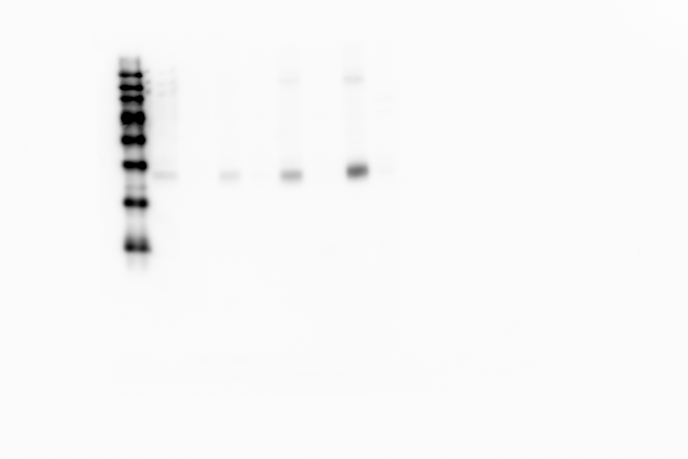

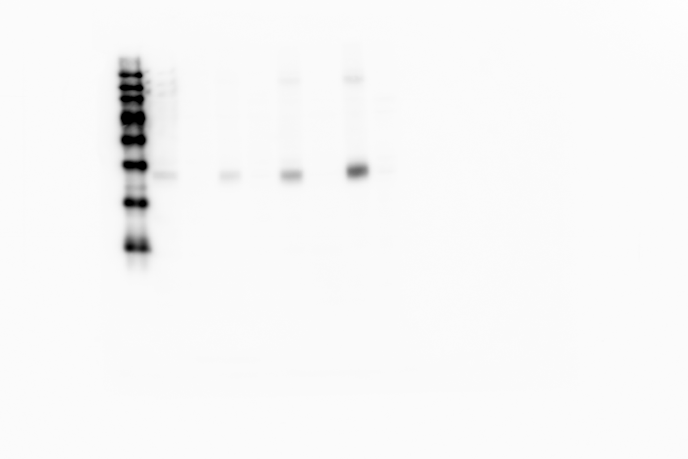

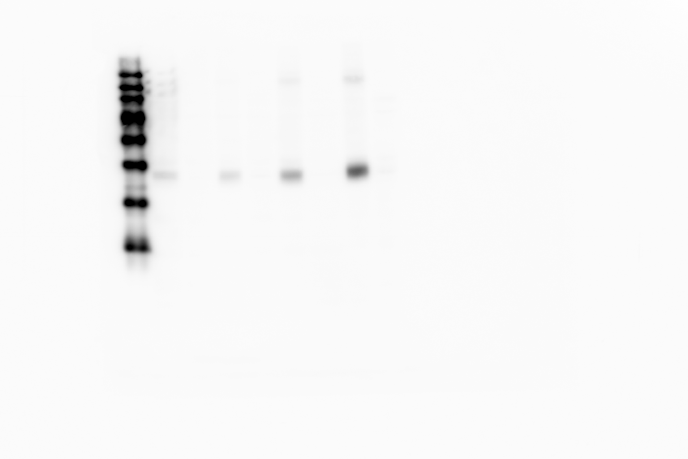

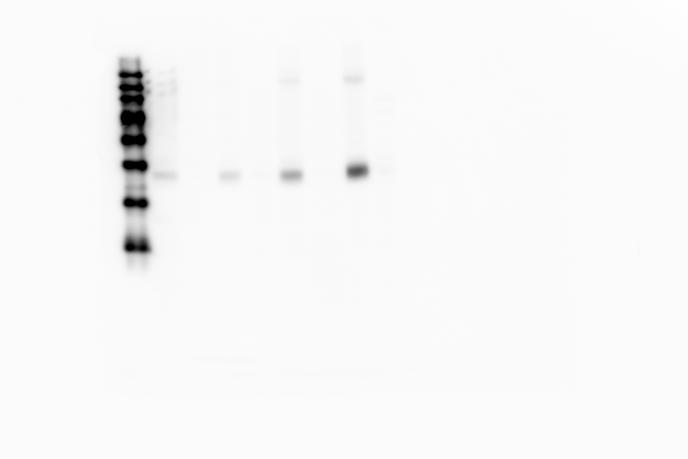

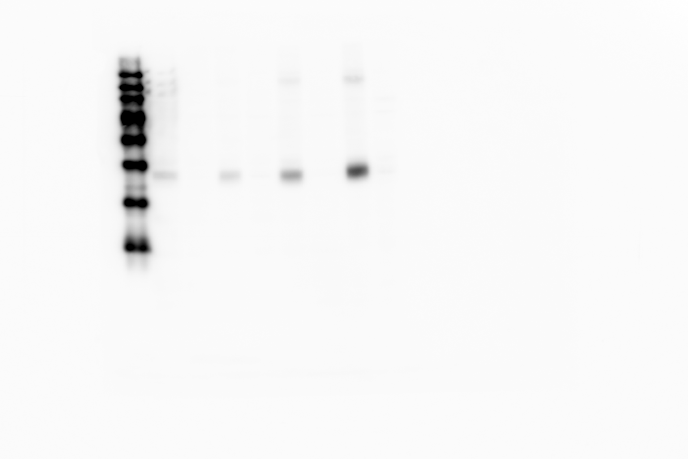

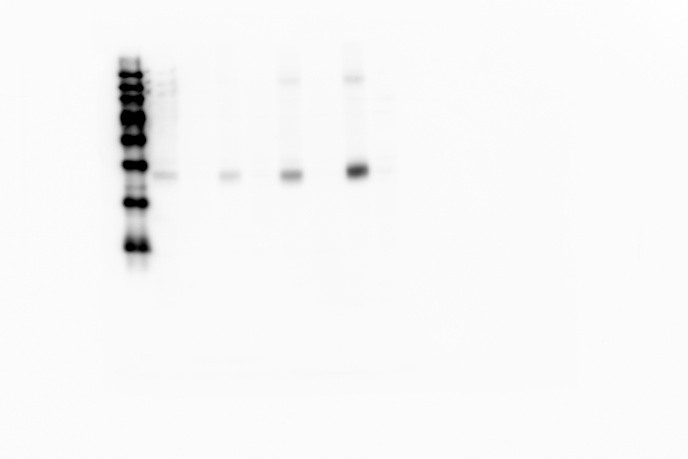


d (8-16)


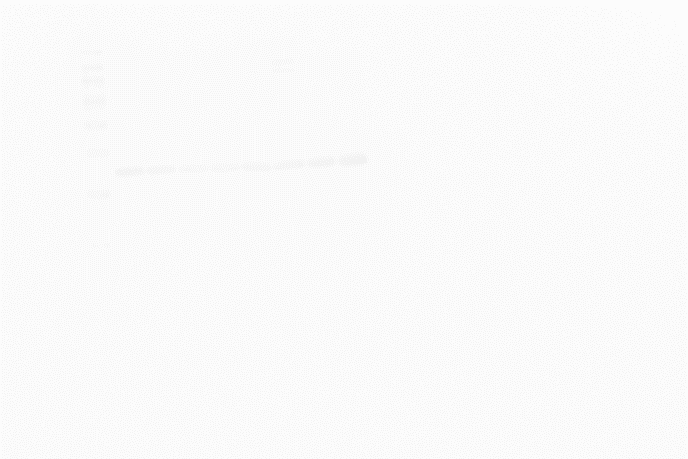

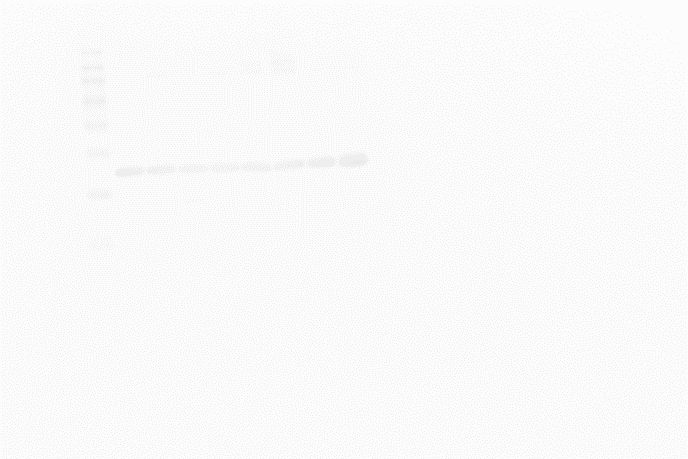

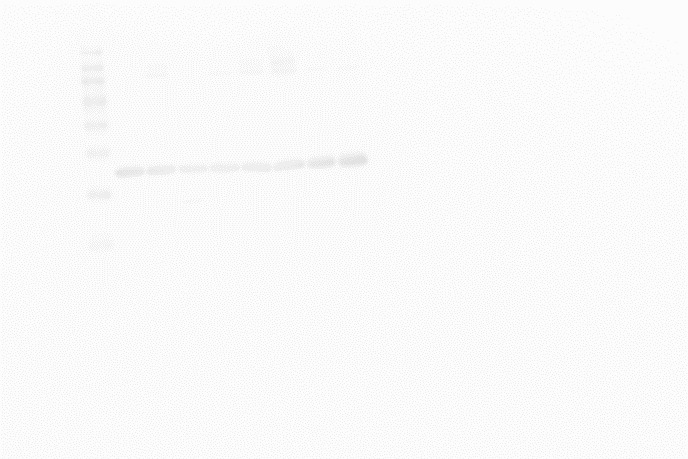

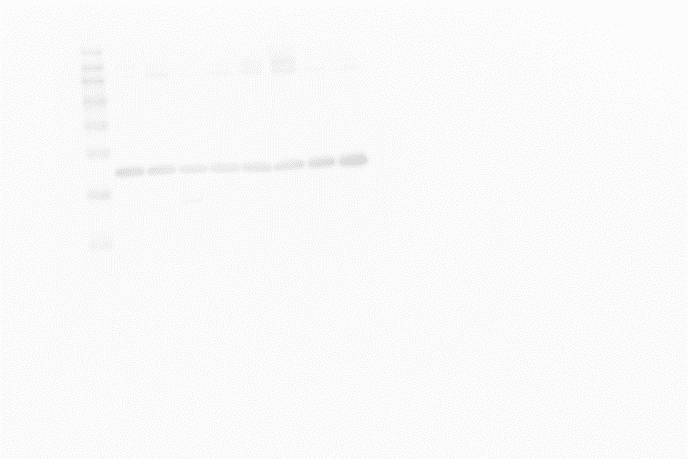

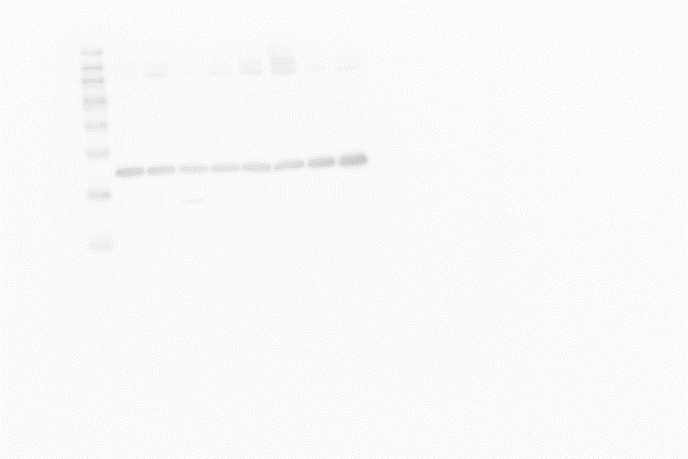

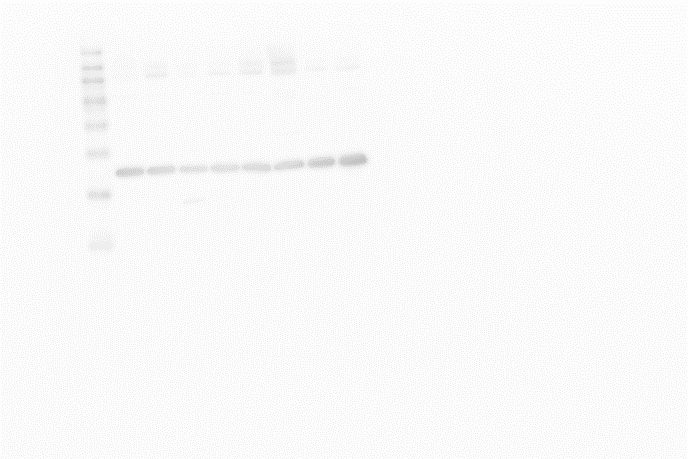

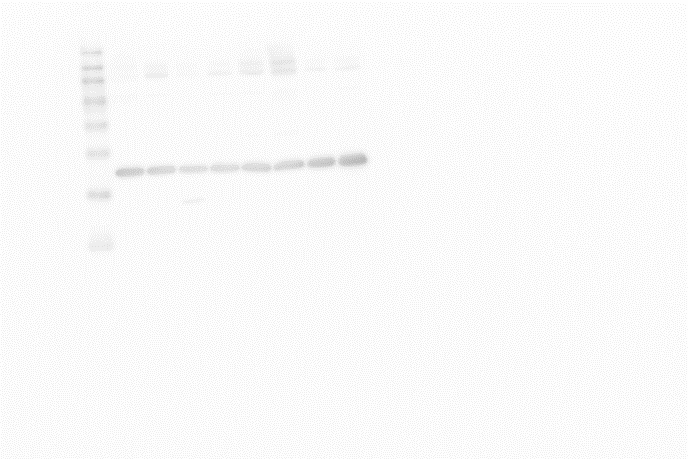

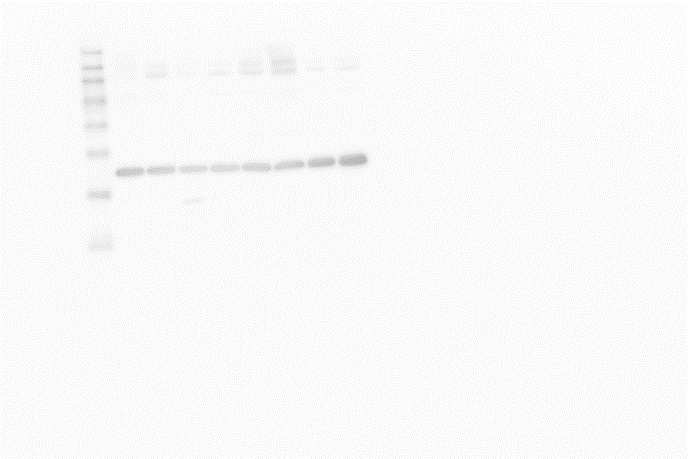


e (1-8)

**Suppl. Figure 3.**
